# Application of three statistical approaches to explore effects of dietary intake of multiple persistent organic pollutants on ER-positive breast cancer risk in the French E3N cohort

**DOI:** 10.1038/s41598-025-85438-9

**Published:** 2025-01-15

**Authors:** Pauline Frenoy, Ismaïl Ahmed, Chloé Marques, Xuan Ren, Gianluca Severi, Vittorio Perduca, Francesca Romana Mancini

**Affiliations:** 1 Inserm, Gustave Roussy, Centre for Research in Epidemiology and Population Health (CESP), “Exposome, Heredity, Cancer, and Health” Team, Université Paris-Saclay, UVSQ, 12 Avenue Paul Vaillant Couturier, 94805 Villejuif, France; 2 Inserm, CESP, Université Paris-Saclay, UVSQ, Villejuif, France; 3 Department of Statistics, Computer Science, Applications “G. Parenti”, University of Florence, Florence, Italy; 4 CNRS, MAP5, Université Paris Cité, F-75006 Paris, France

**Keywords:** Persistent organic pollutants, Multicollinearity, Breast cancer, Cancer epidemiology, Epidemiology

## Abstract

Persistent organic pollutants (POPs) are a group of organic chemical compounds. Contradictory results have emerged in epidemiological studies attempting to elucidate their relationship with breast cancer risk. This study explored the relationship between dietary exposures to multiple POPs and ER-positive breast cancer risk in the French E3N cohort study, using three different approaches to handle multicollinearity among exposures. Intakes of 81 POPs were estimated using food consumption data from a validated semi-quantitative food frequency questionnaire and food contamination data. In the first approach, hierarchical clustering was performed to identify clusters of correlated POPs. For each cluster, the levels of POPs belonging to it were averaged. These average levels were then included in a Cox model to estimate their associations with ER-positive breast cancer occurrence. The second and third approaches applied in the present study were Principal component Cox regression (PCR-Cox) and partial least squares Cox regression (PLS-Cox) respectively, both being dimension-reduction methods (respectively unsupervised and supervised) coupled to a Cox model, used to identify principal components of POPs and to estimate their associations with ER-positive breast occurrence. All models were adjusted for potential confounders previously identified using a directed acyclic graph. The study included 66,722 women with a median follow-up of 20.3 years, during which 3,739 developed an incident ER-positive breast cancer. The variable clustering method did not identify any association between the averaged variables and ER-positive breast cancer risk. Five components were retained using both the PCR-Cox and PLS-Cox methods explaining 82% and 77% of the variance in the initial exposure matrix respectively. Among these components, none was significantly associated with the occurrence of ER-positive breast cancer. This study provides an illustrative example of the application of three distinct statistical methods in the context of highly correlated environmental exposures, discussing their potential relevance and limitations within this specific framework.

**Supplementary Information:**

The online version contains supplementary material available at 10.1038/s41598-025-85438-9.

## Introduction

In 2020, 2.3 million women were diagnosed with breast cancer, making it the most diagnosed of all cancer worldwide^1^. Western countries, and more recently low and middle-income countries, have seen the incidence of breast cancer increase in recent decades^1,2^. Despite a large body of research, the established risk factors are still insufficient to explain its occurrence^3–5^. Environmental exposures to chemical contaminants, particularly contaminants with endocrine-disrupting properties, are suspected of being breast cancer risk factors^6^.

Persistent organic pollutants (POPs) are a group of organic chemical substances sharing some common properties. Due to their resistance to degradation and their potential for long-range transport, they are widespread and persist for long periods of time in the environment. As they can bioaccumulate in the fatty tissue of living organisms, they tend to biomagnify along the food chain, and as such, diet represents the main source of exposure for human populations^7–11^. To protect human health and the environment from their potential negative effects, the Stockholm Convention, an international treaty that aims to decrease their production, use, and releases, entered into force in 2004^12^.

POPs are suspected to exert estrogenic effects, and some of them have demonstrated potential mechanisms of estrogen receptor dysregulation^13–17^. Some POPs have even shown the capacity to promote the proliferation of breast cancer cells in vitro, particularly those tumors that present estrogen receptors (ER-positive)^18,19^. However, when it comes to epidemiological studies attempting to elucidate the relationship between exposure to POPs and breast cancer risk in women, contradictory results have emerged^20–24^. The different approaches to assess POP exposures used in these studies may contribute to explain discrepancies in results. In fact, it is possible to use direct measurements of POPs in biological matrices such as blood or adipose tissue, or indirect estimates of exposure (occupational, airborne, etc.).

In addition, most epidemiological studies estimate the health effects of exposure to individual substances separately. On one hand, such studies may be prone to confounding issues since subjects are simultaneously exposed to a variety of chemicals. On the other hand, attempting to mutually adjust for all exposures using multiple regression models can lead to multicollinearity issues, because exposures to these substances are in general highly correlated, resulting in unstable and unreliable coefficient estimates. To address these challenges when dealing with data on multiple and highly correlated environmental exposures, various statistical approaches exist, each addressing different specific research questions^25^. Namely, in this work we study whether joint exposure to highly correlated pollutants has an effect on outcome, if the patterns of POPs most frequently observed in our study population have an effect on outcome, and explore the patterns of POPs most related to the outcome.

Unsupervised dimension reduction and clustering methods are commonly used in epidemiology when dealing with highly correlated exposures. Variable clustering is a method of hierarchical clustering of exposure variables that can be used to identify distinct clusters of correlated variables. The idea is to group together exposure variables whose individual effects cannot be distinguished due to their high correlations. A variable representing each cluster, typically the average of all the variables that make up the cluster, can then be calculated. These summary statistics can then be incorporated into regression models to estimate their effects. The effect estimated for the summary statistic of a given cluster may be due to one or more exposures belonging to the cluster. Principal component regression (PCR) is a combination of a classical non-supervised dimension reduction technique called principal component analysis (PCA) and a regression model. PCA identifies a reduced set of uncorrelated principal components, which are weighted linear combinations of the original exposure variables, explaining as much as possible the variance in the initial exposure matrix. Subsequently, these principal components can be incorporated into regression models to estimate their effects, Cox models in this work. The identification of the principal components solely relies on the correlation matrix between the exposure variables, disregarding any link between the exposure variables and the outcome. Supervised dimension reduction methods such as Partial least square regression (PLS) are less commonly used in epidemiology. PLS takes into account the relation between the exposures and the outcome when generating the principal components. More precisely, PLS combines a dimension reduction method with a regression model, to identify a set of principal components maximizing the covariance with the outcome, and estimate their association with the outcome^25,26^. This method therefore makes it possible to identify weighted linear combinations of the original exposure variables linked to the outcome of interest. In this work we applied a version of PLS for Cox models. The effects of the combinations identified by PCR and PLS can be thought of as representing the weighted cumulative effects of the original exposures on the outcome of interest.

The objective of the present study was to explore the relations between dietary exposures to multiple POPs and ER-positive breast cancer risk in the French E3N cohort study (*Etude Epidémiologique auprès de femmes de la Mutuelle Générale de l’Education Nationale*), using different approaches to handle multicollinearity among exposures: variable clustering, PCR and PLS.

## Materials and methods

### The E3N cohort

The E3N study, initiated in France in 1990, is an ongoing prospective cohort including only women. The detailed protocol has been previously documented^27,28^. In summary, the study included 98,995 women born between 1925 and 1950 and covered by the French national health insurance plan for people working for the national education system, the *Mutuelle Générale de l’Education Nationale* (MGEN). These women were surveyed through self-administered questionnaires sent every two or three years. A good participation rate of approximately 83% has been maintained over time. The study received approval from the French National Commission for Data Protection and Privacy, and all participants provided written informed consent.

### Assessment of food consumption

Dietary information was gathered using a previously validated semi-quantitative food frequency questionnaire sent in 1993^29^. This questionnaire included questions concerning 208 food items, assessing participants’ typical diet over the preceding year. The questionnaire captured details regarding the consumption of various foods and beverages across eight different occasions: breakfast, morning snack, aperitif before lunch, lunch, afternoon snack, pre-dinner aperitif, dinner and after-dinner snack. To estimate the average daily nutrient intake of the participants, the food composition table derived from the French Information Center on Food Quality (CIQUAL) was used^30^.

### Assessment of dietary exposure to POPs

Data on food contamination were acquired from the second French Total Diet Study (TDS2), conducted by the French Agency for Food, Environmental and Occupational Health and Safety (ANSES)^7,8,31^. In brief, a total of 20,280 distinct food products were purchased from June 2007 to January 2009 in eight regions of France, resulting in 1,352 composite samples of foods prepared as they are usually consumed and analyzed to measure the contamination levels of 445 substances^31^. To handle non-detected or non-quantified values, a lower-bound scenario was employed: values below the limit of detection were replaced with 0, and values below the limit of quantification were replaced with the limit of detection if available, or with 0 otherwise.

Subsequently, the E3N database on food consumption data and the ANSES database on food contaminant concentrations were joined, as explained in detail elsewhere^32^. For each participant, the daily average dietary intake of each substance was estimated by multiplying the mean daily quantities consumed of each food component by the corresponding levels of contamination.

Among the 445 substances initially analyzed in the TDS2 study, 81 POPs were included in the current study:

- 17 congeners of polychlorinated dibenzo-p-dioxins (or dioxins) and polychlorinated dibenzofurans (or furans): 2,3,7,8-tetrachlorodibenzo-p-dioxin (TCDD-2378), 1,2,3,7,8-pentachlorodibenzo-p-dioxin (PCDD-12378), 1,2,3,4,7,8-hexachlorodibenzo-p-dioxin (HCDD-123478), 1,2,3,6,7,8-hexachlorodibenzo-p-dioxin (HCDD-123678), 1,2,3,7,8,9-hexachlorodibenzo-p-dioxin (HCDD-123789), 1,2,3,4,6,7,8-heptachlorodibenzo-p-dioxin (HCDD-1234678), octachlorodibenzodioxin (OCDD), 2,3,7,8-tetrachlorodibenzofuran (TCDF-2378), 1,2,3,7,8-pentachlorodibenzofuran (PCDF-12378), 2,3,4,7,8-pentachlorodibenzofuran (PCDF-23478), 1,2,3,4,7,8-hexachlorodibenzofuran (HCDF-123478), 1,2,3,6,7,8-hexachlorodibenzofuran (HCDF-123678), 2,3,4,6,7,8-hexachlorodibenzofuran (HCDF-234678), 1,2,3,7,8,9-hexachlorodibenzofuran (HCDF-123789), 1,2,3,4,6,7,8-hexachlorodibenzofuran (HCDF-1234678), 1,2,3,4,7,8,9-hexachlorodibenzofuran (HCDF-1234789), octachlorodibenzofuran (OCDF);
- 18 polychlorinated biphenyls (PCBs): 12 dioxin-like congeners (PCB-77, 81, 105, 114, 118, 123, 126, 156, 157, 167, 169 and 189) and 6 non-dioxin-like congeners (PCB-28, 52, 101, 138, 153, 180);
- 12 per- and polyfluoroalkyl substances (PFASs): perfluorodecanoic acid (PFDA), perfluorododecanoic acid (PFDoA), perfluoroheptanoic acid (PFHpA), perfluorohexanoic acid (PFHxA), perfluorononanoic acid (PFNA), perfluorooctanoic acid (PFOA), perfluorotetradecanoic acid (PFTeDA), perfluorotridecanoic acid (PFTrDA), perfluoroundecanoic acid (PFUnA), perfluorobutanesulfonic acid (PFBS), perfluorohexanesulfonic acid (PFHxS), perfluorooctanesulfonic acid (PFOS);
- 14 brominated flame retardants (BFRs): 8 polybrominated diphenyl ether congeners (PBDE-28, 47, 99, 100, 153, 154, 183, 209), 3 polybrominated biphenyl congeners (PBB-52, 11, 153) and 3 hexabromocyclododecane congeners (HBCD-α, β, γ);
- 20 polycyclic aromatic hydrocarbons (PAHs): anthracene (AN), benzo[a]anthracene (BaA), benzo[a]pyrene (BaP), benzo[b]fluoranthene (BbF), benzo[c]fluorine (BcFL), benzo[g, h,i]perylene (BghiP), benzo[j]fluoranthene (BjF), benzo[k]fluoranthene (BkF), chrysene (CHR), cyclopenta[c, d]pyrene (CPP), dibenzo[a, h]anthracene (DBahA), dibenzo[a, e]pyrene (DbaeP), dibenzo[a, h]pyrene (DbahP), dibenzo[a, i]pyrene (DbaiP), dibenzo[a, l]pyrene (DbalP), fluoranthene (FA), indeno[1,2,3-cd]pyrene (IP), 5-methylchrysene (MCH), phenanthrene (PHE) and pyrene (PY).

### Identification of breast cancer and death

The occurrence of breast cancer was identified mainly by self-reporting in questionnaires sent every two or three years. Additional cases were identified from next-of-kin spontaneous reports and information from the national cause of death registry. Pathology reports or medical records were obtained for 93% of cases, enabling diagnosis validation. The estrogen receptor (ER) status of the cancer (positive or negative) was obtained from the pathology report. This study focused on validated cases of ER-positive breast cancer.

Information on deaths came from the MGEN health insurance database, family member declarations, and the national cause of death registry.

### Covariates

Adjustment variables included in the final Cox models described below were selected using a directed acyclic graph (DAG) to identify the total effect of dietary intake of POPs on breast cancer risk (Supplementary Fig. 1), developed using the online software DAGitty^33^. To ensure temporal consistency, adjustment covariate values were obtained from the first and second E3N questionnaires (sent in June 1990 and January 1992, respectively) whenever available, and from the third E3N questionnaire otherwise (sent in June 1993). This approach ensured that most of the covariate values preceded the collection of the primary exposure variables via the dietary questionnaire (sent in June 1993), which provided information on food consumption during the preceding year.

Information on school education level was collected through the initial questionnaire sent in 1990. Information on smoking status, body mass index (BMI, obtained from height and weight), parity and age at first full-term pregnancy (FFTP), cumulated duration of previous breastfeeding, previous use of contraceptive pill, menopausal status, and recent use of menopausal hormone therapy (MHT) were collected through the second questionnaire sent in 1992. Finally, the questionnaire sent in 1993 provided information on physical activity and eating habits. More specifically, a previously validated food frequency questionnaire was used to collect the usual frequency and quantity of food and drink consumed, using a standardized illustrated booklet. Detailed description of the questionnaire has been previously published^29^. To estimate the participants’ daily intakes of alcohol (in g of ethanol/day), lipids (in g/day) and calories (in kcal/day), the food composition table derived from the French food composition table of the French Information Center on Food Quality (CIQUAL) was used^30^. Adherences to both the western and prudent dietary patterns, derived from PCA, were also determined using the dietary questionnaire, as described elsewhere^34^. Adherence to French dietary guidelines was estimated using the simplified “Programme National Nutrition Santé - guidelines score 2” (sPNNS-GS2)^35,36^.

Covariates with 5% or less of missing values were imputed using the mode for categorical variables and the median for continuous variables. For covariates with more than 5% of missing values (i.e., recent use of MHT and BMI), a missing category was created.

### Study population

All women who had filled out the dietary questionnaire distributed in June 1993 were eligible for the current study. Women with a prevalent cancer diagnosis at baseline, those who did not complete any subsequent questionnaires following the dietary questionnaire, those who had reported extreme energy intake values (i.e., below the 1st or above the 99th percentiles for the ratio of energy intake to energy requirement) and breast cancer cases having missing status for the ER were excluded.

### Statistical analyses

Baseline characteristics (mean and standard deviation for continuous variables, numbers, and proportions for categorical variables) of the study population were described in the overall population. Number and proportion of missing data were also described, if any. A heatmap representing Spearman rank correlation coefficients between dietary intake of the 81 POPs was constructed.

Three statistical approaches were used to explore the relation between dietary exposure to POPs and ER-positive breast cancer occurrence. For all three approaches, the predictor matrix was composed of the estimated dietary exposure to the 81 POPs, which were centered and reduced. The response variable was the time of occurrence of ER-positive breast cancer.

The first approach used was a variable clustering method followed by Cox regression models (Varclus-Cox). A hierarchical cluster analysis on the 81 variables of exposures to POPs was performed to identify clusters of highly correlated POPs, using squared Pearson correlations as similarity measures and the complete-linkage clustering algorithm implemented in the varclus function of the Hmisc R package (version 5.1-1). To determine the number of clusters to retain, we first identified elbows on the graph representing the dissimilarity of the two clusters being grouped at each step of the algorithm (the height). Among these candidates, we chose a number of clusters corresponding to a good compromise between sufficient high correlations among POPs within each cluster as well as sufficient low correlations between the cluster summary statistics (their means). This ensures that that a single variable can correctly represent each cluster, and that it is possible to estimate their effects in a Cox model without multicollinearity issues. The compactness and separation of the selected clusters was assessed using the silhouette measure. Then, the summary statistics of each cluster were all included in the same Cox regression model adjusted on potential confounders.

The second approach was principal component Cox regression (PCR-Cox). A PCA was performed to identify linear combinations of POPs explaining as much as possible of the variance of the original exposure matrix, using the PCA function of the FactoMineR package (version 2.9). To determine the number of principal components to retain, we considered the variance explained by each principal component, along with their interpretability, based on considerations on the loadings (i.e., the coefficients of the original exposures in the linear combinations defining the principal components). The retained principal components were then included in a Cox regression model adjusted on potential confounders.

The third approach, partial least square Cox regression (PLS-Cox), was used to identify linear combinations of POPs associated with the occurrence of ER-positive breast cancer^37,38^. The plsRcox function of the plsRcox R package (version 1.7.7) was used. To determine the number of principal components to retain, the variance explained by each component of the initial matrix of exposures was considered, along with the interpretability of the principal components. A second analysis step was conducted to estimate the adjusted effects of the retained principal components on the risk of ER-positive breast cancer, using a Cox regression model adjusted on potential confounders. These two steps were conducted on two different datasets to avoid post-selection inference issues. Therefore, the study population was randomly separated into two distinct groups: 70% of the study population was used to identify PLS principal components (dataset 1), and the remaining 30% of the population was used to estimate their adjusted effects (dataset 2).

The three approaches used cause-specific Cox proportional hazards model. Age was used as the time scale, and delayed entry was incorporated by considering the age at which the dietary questionnaire was completed as the time at entry. Exit time was the age of diagnosis of ER-positive breast cancer (for cases), the age at the last completed questionnaire before death or lost to follow-up, the age at diagnosis of another cancer, or the age at the end of the follow-up period (November 2014), whichever occurred first. For the identification of principal components with the PLS-Cox method, time-on-study was used as the time scale. Indeed, using age as the time scale was not feasible because the PLS-Cox function did not support delayed entry. Subsequently, a final Cox model was fitted to estimate the effect of the principal components using age as the time-scale.

Covariates identified by the DAG depicted in supplementary Fig. 1 were included in the model. Thus, the main models were stratified on birth generation (in 5 categories: ≤1930; (1930–1935]; (1935–1940]; (1940;1945]; >1945), and were adjusted for school education level (< 12 years of studies; 12 to 14 years of studies; >14 years of studies), smoking status (non-smoker; former smoker; current smoker), body mass index (< 18.5; [18.5–22.5); [22.5–25); [25–30); ≥30 kg/m^2^; missing values), parity and age at FFTP (nulliparous; one or two children and age at FFTP < 30 years; more than two children and age at FFTP < 30 years; age at FFTP ≥ 30 years), cumulated duration of previous breastfeeding (no breastfeeding: less than 6 months of breastfeeding; at least 6 months of breastfeeding), utilization of contraceptive pill (ever; never), menopausal status and recent use of MHT (pre-menopaused, menopaused with recent use of MHT, i.e. less than a year ago; menopaused without recent use of MHT; menopaused and missing data on recent use of MHT), physical activity (continuously in metabolic equivalents of task-hour/week), daily alcohol intake (continuously in g of ethanol/day), daily lipids intake (continuously in g/day) and daily total energy intake except from alcohol and lipid (continuously in kcal/day). Birth generation has been categorized to allow a stratification of the baseline hazard, because we assume the effect of this variable is likely to be non-proportional^39^. BMI has been categorized because its effect is likely to be non-linear, and to allow the introduction of a missing data category. Other quantitative covariates were kept continuous and were introduced with linear terms in the main analyses. Compliance with the proportional hazard assumption was assessed for each covariate in the final Cox models for each approach by displaying graphs of the Schoenfeld residuals. Finally, for greater clarity, the formulae for the final Cox models fitted to each of the three approaches, fitted after identifying the final numbers of clusters and principal components, are presented in the annex 1 of the supplementary materials.

All statistical tests were two-sided, and the threshold for statistical significance was set at 5%. Statistical analyses were performed using the Statistical Analysis Systems software, version 9.4 (SAS Institute, Cary, NC), and the R software, version 4.0.3.

### Sensitivity analyses

To explore the possibility of residual confounding due to diet (supplementary Fig. 1), the main models were separately further adjusted on adherence to prudent and western dietary patterns on the one hand, and on adherence to French dietary guidelines on the other hand. To reduce the impact of a potential reverse causality bias and to explore the long-term effects of the exposures, a 5-year time lag was introduced between exposure assessment and the start of follow-up, i.e. we started the follow-up 5 years after the response to the dietary questionnaire. Given the large number of covariates included in the main model, a more parsimonious adjustment set was tested to assess possible overfitting. For the latter, the adjustment covariates for which the p-value of the association with breast cancer risk was < 0.10 were selected, i.e. school education level, parity and age at FFTP, menopausal status and recent use of MHT, daily alcohol intake and daily lipids intake. Because we suppose that continuous main exposures and adjustment variables may have non-linear effects on the outcome, a sensitivity analysis modeling all continuous variables with natural cubic splines with 3 degrees of freedom was performed, except for the PLS principal components. For the latter, we kept linear terms because these variables were constructed to be linearly associated with the outcome. Finally, to explore a possible lack of power in the identification of the effects of principal components identified by PLS-Cox using the dataset 2, we tried to run the same model on the entire dataset.

## Results

### Selection of the study population

Among the 74,522 women who had filled out the dietary questionnaire, 4,709 women with a prevalent cancer diagnosis at baseline, 568 with no questionnaire completed after the dietary questionnaire, 1,366 with extreme energy intake values reported, and 1,157 incident breast cancer cases with missing status for the ER were excluded. Ultimately, the study sample consisted of 66,722 women among which 3,739 developed an ER-positive incident breast cancer during the follow-up.

### Characteristics of the study population

The study population was constituted of 66,722 women followed for a maximum of 21.4 years (from 1993 to 2014). The median duration of follow-up was 20.3 years, and the total duration of follow-up was 1,184,133 person-years. Characteristics of the study population are presented in Table 1.

**Table 1:** Baseline or pre-baseline characteristics of the study population (*N* = 66,722).

	Mean (Standard deviation) or Number (Percent)
**Socio-demographic characteristics**	
Age at baseline (years)	52.8 (6.65)
Birth generation	
≤1930	6,414 (9.61%)
(1930; 1935]	8,931 (13.39%)
(1935; 1940]	13,354 (20.01%)
(1940; 1945]	16,459 (24.67%)
>1945	21,564 (32.32%)
School education level (years) ^(1)^	
<12	7,524 (11.28%)
[12–14]	33,036 (49.51%)
>14	23,890 (35.81%)
Missing value	2,272 (3.41%)
**Lifestyle and reproductive characteristics**	
Smoking status ^(2)^	
Current	8,464 (12.69%)
Former	21,312 (31.94%)
Never	34,379 (51.53%)
Missing value	2,567 (3.85%)
Menopausal status and recent menopausal hormone therapy (MHT) use ^(2)^	
Premenopausal	35,200 (52.76%)
Menopaused and recent MHT use (less than a year ago)	9,444 (14.15%)
Menopaused and no recent MHT use	18,859 (28.27%)
Menopaused and missing value on recent MHT use	3,219 (4.82%)
Parity ^(2)^	
Nulliparous	7,788 (11.67%)
One or two children	38,945 (58.37%)
More than two children	19,982 (29.95%)
Missing value	7 (0.01%)
Age at first full-term pregnancy, for non-nulliparous women ^(2)^	
<30 years	51,040 (86.62%)
≥30 years	7,061 (11.98%)
Missing value	826 (1.40%)
Cumulative duration of previous breastfeeding ^(2)^	
No breastfeeding	25,246 (37.84%)
Cumulative duration of breastfeeding < 6 months	26,105 (39.13%)
Cumulative duration of breastfeeding ≥ 6 months	12,243 (18.35%)
Missing value	3,128 (4.69%)
Contraceptive pill use (current or past) ^(2)^	
Never	25,113 (37.64%)
Ever	39,179 (58.72%)
Missing value	2,430 (3.64%)
Body mass index (kg/m^2^) ^(2)^	22.75 (3.20)
<18.5	2,431 (3.64%)
[18.5–22.5)	31,972 (47.92%)
[22.5–25)	16,750 (25.10%)
[25–30)	9,903 (14.84%)
≥30	2,038 (3.05%)
Missing value	3,628 (5.44%)
Total physical activity (metabolic equivalents of task hours per week) ^(3)^	49.23 (49.72)
Missing value	433 (0.65%)
**Dietary consumptions**	
Lipid consumption (g/day) ^(3)^	89.02 (27.02)
Alcohol consumption (g of ethanol/day) ^(3)^	11.58 (13.91)
Total energy intake (kcal/day) ^(3)^	1328.90 (356.03)

(1) Information collected at the first questionnaire sent in 1991.

(2) Information collected at the second questionnaire sent in 1992.

(3) Information collected at the third questionnaire sent in 1993.

Correlations between dietary intake of POPs are presented in the supplementary Fig. 2. High Spearman correlation coefficients were observed especially for POPs belonging to the same family of chemicals. For example, Spearman rank correlation coefficients between PCBs were comprised between 0.67 and 1.

### Associations between dietary exposure to mixtures of POPs and ER-positive breast cancer risk

We retained 11 clusters with the Varclus-Cox approach (i.e., the clusters found at the iteration number 70 of the hierarchical clustering algorithm, corresponding to a total height of 0.59), each containing between 1 and 28 POPs. Supplementary Figs. 3, 4 and 5 represent the corresponding elbow plot, the dendrogram and the heatmap of the dietary exposures to POPs, with the clusters framed. The average silhouette measure was 0.41 (Supplementary Fig. 6). The cluster memberships of each POPs, and the range of correlations between each pair of POPs within each cluster are presented in supplementary Table 1: considering all clusters, these correlations ranged from 0.46 to 1. No significant adjusted association between the summary statistics of each cluster and ER-positive breast cancer risk has been identified by Cox models (Table 2). The graphs representing the Schoenfeld residuals assessing the proportional hazards assumption for each variable of the model are presented in supplementary Fig. 7. The hypothesis appeared to be met for all variables.

**Table 2:** Association between summary statistics of each cluster of POPs obtained by hierarchical variable clustering and ER-positive breast cancer risk in the E3N cohort (*N* = 66,722).

	AP0: Unadjusted		AP1: Adjusted	
	HR [95% CI]	p-value	HR [95% CI]	p-value
Cluster 1 summary statistic, for 1 SD increase	**1.06 [1.03–1.09]**	**< 0.001**	1.04 [0.97–1.12]	0.219
Cluster 2 summary statistic, for 1 SD increase	1.03 [0.99–1.06]	0.111	0.96 [0.91–1.02]	0.164
Cluster 3 summary statistic, for 1 SD increase	1.02 [0.99–1.05]	0.184	0.99 [0.93–1.06]	0.856
Cluster 4 summary statistic, for 1 SD increase	**1.05 [1.01–1.08]**	**0.005**	1.00 [0.92–1.10]	0.923
Cluster 5 summary statistic, for 1 SD increase	0.98 [0.95–1.02]	0.325	0.99 [0.96–1.02]	0.489
Cluster 6 summary statistic, for 1 SD increase	1.02 [0.98–1.05]	0.318	0.99 [0.96–1.03]	0.647
Cluster 7 summary statistic, for 1 SD increase	**1.04 [1.01–1.08]**	**0.004**	1.03 [0.98–1.08]	0.281
Cluster 8 summary statistic, for 1 SD increase	**1.04 [1.01–1.07]**	**0.008**	1.00 [0.95–1.05]	0.972
Cluster 9 summary statistic, for 1 SD increase	**1.04 [1.01–1.07]**	**0.021**	1.00 [0.96–1.05]	0.981
Cluster 10 summary statistic, for 1 SD increase	1.01 [0.98–1.04]	0.537	0.98 [0.94–1.02]	0.243
Cluster 11 summary statistic, for 1 SD increase	1.03 [1.00–1.06]	0.082	1.02 [0.98–1.05]	0.404

Hazard ratios (HR) and 95% confidence interval (CI) are estimated by Cox univariable and multivariable regression models. Values in bold are significant (i.e., p<0.05).

SD: standard deviation.

AP0: Use of age as the time-scale (years), without stratification or adjustment variables.

AP1: Use of age as the time-scale (years), stratification of the baseline hazard on birth generation (≤ 1930; (1930–1935]; (1935–1940]; (1940;1945]; >1945), adjustment on school education level (< 12 years; 12 to 14 years; >14 years), smoking status (non-smoker; former smoker; current smoker), body mass index (< 18.5; [18.5–22.5); [22.5–25); [25–30); ≥30 kg/m2; missing values), parity and age at FFTP (nulliparous; one or two children and age at FFTP < 30; more than two children and age at FFTP < 30; age at FFTP ≥ 30), cumulated duration of previous breastfeeding (no breastfeeding: less than 6 months of breastfeeding; at least 6 months of breastfeeding), utilization of contraceptive pill (ever; never), menopausal status and recent use of MHT (pre-menopaused, menopaused with recent use of MHT; menopaused without recent use of MHT, menopaused and missing data on recent used of MHT), physical activity (continuously in metabolic equivalents of task-hour/week), daily alcohol intake (continuously in g of ethanol/day), daily lipids intake (continuously in g/day), and daily total energy intake except from alcohol and lipid (continuously in kcal/day), and mutual adjustment of each cluster summary statistic to each other.

With the PCR-Cox approach, we kept 5 principal components explaining 82.0% of the initial exposure matrix variance. The loadings of POPs in these principal components are presented in supplementary Table 2. The first principal component explained 51.3% of the variance and was characterized by positive loadings for all chemicals, particularly strong for PCBs, dioxins & furans, and BFRs. The other principal components explained 12.5%, 7.8%, 6.0% and 4.4% of the variance. No significant adjusted association between these principal components and ER-positive breast cancer risk has been identified by Cox models (Table 3). On inspection of the Schoenfeld residual plots, the proportional hazards assumption appeared reasonable (supplementary Fig. 8).

**Table 3:** Association between principal components of POPs obtained by PCA and ER-positive breast cancer risk in the E3N cohort (*N* = 66,722).

	AP0: Unadjusted		AP1: Adjusted	
	HR [95% CI]	p-value	HR [95% CI]	p-value
Component 1, for 1 SD increase	**1.05 [1.01–1.08]**	**0.005**	1.02 [0.97–1.07]	0.441
Component 2, for 1 SD increase	**1.04 [1.01–1.08]**	**0.015**	1.03 [0.98–1.07]	0.211
Component 3, for 1 SD increase	1.01 [0.98–1.04]	0.659	1.00 [0.97–1.04]	0.776
Component 4, for 1 SD increase	0.98 [0.95–1.01]	0.203	0.99 [0.96–1.02]	0.487
Component 5, for 1 SD increase	1.00 [0.97–1.04]	0.786	1.00 [0.97–1.03]	0.965

Hazard ratios (HR) and 95% confidence interval (CI) are estimated by Cox multivariable regression models. Values in bold are significant (i.e., p<0.05).

SD: standard deviation.

AP0: Use of age as the time-scale (years), without stratification or adjustment variables.

AP1: Use of age as the time-scale (years), stratification of the baseline hazard on birth generation (≤ 1930; (1930–1935]; (1935–1940]; (1940;1945]; >1945), adjustment on school education level (< 12 years; 12 to 14 years; >14 years), smoking status (non-smoker; former smoker; current smoker), body mass index (< 18.5; [18.5–22.5); [22.5–25); [25–30); ≥30 kg/m2; missing values), parity and age at FFTP (nulliparous; one or two children and age at FFTP < 30; more than two children and age at FFTP < 30; age at FFTP ≥ 30), cumulated duration of previous breastfeeding (no breastfeeding: less than 6 months of breastfeeding; at least 6 months of breastfeeding), utilization of contraceptive pill (ever; never), menopausal status and recent use of MHT (pre-menopaused, menopaused with recent use of MHT; menopaused without recent use of MHT, menopaused and missing data on recent used of MHT), physical activity (continuously in metabolic equivalents of task-hour/week), daily alcohol intake (continuously in g of ethanol/day), daily lipids intake (continuously in g/day), and daily total energy intake except from alcohol and lipid (continuously in kcal/day), and mutual adjustment of each principal component to each other.

With the PLS-Cox approach, we selected 5 principal components, explaining 77.2% of the variance of the initial exposure matrix. The first principal component explained 50.3% of the variance and was characterized by positive loadings for almost all chemicals, particularly strong for dioxins & furans, and PAHs. The other principal components explained between 3.0% and 11.9% of the variance and were characterized by both positive and negative weights. Supplementary Table 3 shows the loadings of the 81 substances in each principal component. No significant adjusted association between these principal components and ER-positive breast cancer risk has been identified by Cox models (Table 4). The proportional hazard assumption seemed to be respected according to the Schoenfeld residual plots (supplementary Fig. 9).

**Table 4:** Association between principal components of POPs obtained by PLS-Cox and ER-positive breast cancer risk in the E3N cohort (*N* = 20,127).

	AP0: Unadjusted		AP1: Adjusted	
	HR [95% CI]	p-value	HR [95% CI]	p-value
Component 1, for 1 SD increase	**1.06 [1.00–1.13]**	**0.035**	1.06 [0.97–1.17]	0.197
Component 2, for 1 SD increase	0.99 [0.93–1.04]	0.642	0.99 [0.93–1.05]	0.765
Component 3, for 1 SD increase	0.97 [0.92–1.03]	0.307	0.97 [0.91–1.03]	0.256
Component 4, for 1 SD increase	1.01 [0.95–1.07]	0.774	1.01 [0.96–1.07]	0.650
Component 5, for 1 SD increase	**1.07 [1.01–1.13]**	**0.029**	1.05 [0.99–1.11]	0.132

Hazard ratios (HR) and 95% confidence interval (CI) are estimated by Cox multivariable regression models. Values in bold are significant (i.e., p<0.05).

SD: standard deviation.

AP0: Use of age as the time-scale (years), without stratification or adjustment variables.

AP1: Use of age as the time-scale (years), stratification of the baseline hazard on birth generation (≤ 1930; (1930–1935]; (1935–1940]; (1940;1945]; >1945), adjustment on school education level (< 12 years; 12 to 14 years; >14 years), smoking status (non-smoker; former smoker; current smoker), body mass index (< 18.5; [18.5–22.5); [22.5–25); [25–30); ≥30 kg/m2; missing values), parity and age at FFTP (nulliparous; one or two children and age at FFTP < 30; more than two children and age at FFTP < 30; age at FFTP ≥ 30), cumulated duration of previous breastfeeding (no breastfeeding: less than 6 months of breastfeeding; at least 6 months of breastfeeding), utilization of contraceptive pill (ever; never), menopausal status and recent use of MHT (pre-menopaused, menopaused with recent use of MHT; menopaused without recent use of MHT, menopaused and missing data on recent used of MHT), physical activity (continuously in metabolic equivalents of task-hour/week), daily alcohol intake (continuously in g of ethanol/day), daily lipids intake (continuously in g/day), and daily total energy intake except from alcohol and lipid (continuously in kcal/day), and mutual adjustment of each principal component to each other.

### Sensitivity analyses

Additional adjustments for adherence to prudent and western dietary patterns, adherence to French dietary guidelines, the introduction of a 5-year lag between exposure assessment and the start of follow-up and the use of a reduced set of adjustment resulted in similar results to the main analyses (supplementary Tables 4, 5 and 6).

The modeling of all continuous adjustment variables with penalized spline functions led to similar results for the PLS-Cox approach (supplementary Table 7). With the Varclus-Cox and PCR-Cox methods, no exposure variables were significantly associated with the occurrence of ER-positive breast cancer when continuous adjustment and exposure variables were modeled with penalized spline functions. Shapes of the associations and global p-values are presented in supplementary Figs. 10 and 11.

The fitting of the main model on the entire study sample for the PLS-Cox approach led to a reduction in confidence interval width, and to variations of the HRs (higher for components 2, 3 and 4; lower for components 1 and 5) (supplementary Table 7). A significant association was highlighted for the fourth principal component (HR [95%CI]: 1.05 [1.01–1.08]).

## Discussion

This study aimed to explore the potential relationship between dietary exposure to multiple highly correlated POPs and the risk of ER-positive breast cancer. Three different statistical methods, chosen among the most widely used or up-to-date, were applied to deal with multicollinearity: the Varclus-Cox approach, the PCR-Cox approach and the PLS-Cox approach. Each of these methods addresses specific research questions, respectively: Does joint exposure to highly correlated POPs have an effect on outcome? Do the patterns of POPs most frequently observed in our study population have an effect on outcome? What are the patterns of POPs most related to the outcome? And what are their effects?

The Varclus-Cox approach did not identify any significant association between the summary statistics of the 11 identified clusters of correlated POPs and ER-positive breast cancer risk after accounting for potential confounding factors. Principal components of POPs constructed to explain the maximum of variability of the original exposure matrix also exhibited no significant association in the PCR-Cox models. Linear combinations of POPs derived using the PLS-Cox approach, which aimed to capture maximum covariance between the original exposure matrix and the outcome, were also not significantly associated with ER-positive breast cancer risk in the dataset 2.

The absence of significant association with ER-positive breast cancer risk for the summary statistic of a given cluster identified with the Varclus-Cox approach does not necessarily mean that no substance within that cluster has an effect. Indeed, if POPs belonging to the same cluster have opposite effects on the outcome, the estimated effect of the summary statistic can be null. Additionally, the average silhouette score obtained for the retained 11 clusters suggests moderate performance in terms of compactness and separation. This indicates that the identified clusters do not optimally separate pollutants with low correlations, or group highly correlated pollutants. While a higher number of clusters would improve the silhouette score, it would also introduce multicollinearity issues in estimating their associations.

Similarly, for the PCR-Cox method, the absence of associations may be due to the opposing effects of different POPs with non-null loadings, either positive or negative, in the same principal component, resulting in a null overall effect.

Because the PCR-Cox method is not supervised, relevant POPs in relation to breast cancer risk may not have been well represented in the retained weighted linear combinations with this approach. The PLS-Cox method, on the other hand, specifically identifies the most relevant linear combinations of POPs in relation to ER-positive breast cancer risk. However, in the current implementation in R, adjustment variables cannot be taken into account when identifying these principal components. Therefore, high positive or negative loadings could mainly be due to confounding factors rather than representing the real contributions of the corresponding POPs to the effect of the principal component considered.

As the PLS-Cox approach is a supervised method, the study sample had to be divided into two parts to avoid post-selection inference problems. Indeed, testing associations on the same data used to identify the principal components would increase the chances of false positives. Using only part of the study sample to test associations was therefore necessary, but reduced statistical power. To address this limitation, we performed sensitivity analyses on the full sample, which found a significant association. In these analyses, the significant association may be due to over-fitting.

Most epidemiological studies focus on the effects of individual POPs or the sums of POPs belonging to the same family. Concerning their potential effects on ER-positive breast cancer risk, published results are globally inconsistent. A recent review of the literature has highlighted that some individual POPs seem consistently associated with higher breast cancer risk in several studies (such as PCB-118, 138, 170, and 180 measured in blood, and PCB-105 measured in breast adipose tissue), but the studies included in this review had moderate to critical risk of bias^20^. To our knowledge, no other study has included such a large number of POPs and investigated their effects using specific methods to manage multicollinearity, so our results are not easy to compare.

This study has several limitations. The estimation of dietary exposures to POPs was based on dietary consumption data collected in 1993, and on contamination levels measured in food samples purchased between 2007 and 2009. Food contamination levels may have changed between these two periods, potentially leading to inaccurate baseline estimates of dietary POPs intake. Nevertheless, due to the persistent nature of these compounds in the environment, the decrease in contamination levels for the selected POPs is expected to be minimal. The use of food frequency questionnaires to estimate dietary consumption may also have contributed to imperfect estimation of the exposure. This is due to challenges associated with accurately recalling and estimating average food intake over an extended duration, as well as the potential influence of social desirability bias in self-reported consumption. However, because the dietary questionnaire was administered at the baseline, prior to any breast cancer diagnoses, any resulting errors are likely to be non-differential. Moreover, even though specific methods adapted to handle highly correlated variables were employed, it is important to note that if two chemicals are perfectly correlated, no statistical methods would be able to distinguish their effects. Additionally, none of the three methods used allowed for the consideration of potential interactions between different POPs. Despite the large number of chemicals included in this study, residual confounding due to co-exposure to other substances might have arisen. Finally, since the E3N cohort comprises only middle-aged women, and who generally have a higher level of school education compared to the broader population, caution should be exercised when generalizing these results.

This study has also several strengths. This is one of the rare studies investigating the effect of multiple POPs on breast cancer occurrence. In order to deal with multicollinearity issues, three statistical methods were used, adapted with longitudinal data. The combination of three different methods estimating different types of effects allowed to explore the links between dietary exposure to POPs and breast cancer occurrence. Although these methods explored different research questions, their results were broadly consistent, with none of them highlighting any effects of dietary intake of POPs on ER-positive breast cancer risk. Moreover, the long follow-up duration allowed to explore long term health effects of POPs, including a large number of cases. Additionally, the exclusion of prevalent breast cancer cases at baseline ensures that dietary habits were evaluated prior to any breast cancer diagnoses, preventing from a potential reverse causation bias. The validation of the food frequency questionnaire and of all breast cancer cases has ensured the use of good quality data for this study. Moreover, the wealth of data accessible from the E3N cohort made it possible to adjust the estimates on numerous covariates, which were chosen using a DAG. Finally, the STROBE-nut reporting guidelines for observational studies in nutritional epidemiology were followed (supplementary Table 8)^40^.

This study did not identify any effect of dietary exposure to POPs on ER-positive breast cancer occurrence when applying three different statistical approaches. However, we believe that this work can be informative for future epidemiological studies dealing with highly correlated environmental exposure variables. Indeed, in this context, a variety of statistical methods exist addressing different types of research questions, each of which presents advantages and drawbacks^25^. By providing an illustrative example of the application of three distinct statistical methods in the presence of highly correlated environmental exposures, the present study has discussed their potential relevance and limitations in this context.

## Electronic supplementary material

Below is the link to the electronic supplementary material.

Supplementary Material 1.

## Data Availability

The datasets analysed during the current study are available from the corresponding author on reasonable request.
