# Supplementary material for: Application of three statistical approaches to explore effects of dietary intake of multiple persistent organic pollutants on ER-positive breast cancer risk in the French E3N cohort
Source: Sci Rep. 2025 Jan 15;15:2058. doi: 10.1038/s41598-025-85438-9 (PMC11735606; doi:10.1038/s41598-025-85438-9)
Supplement: Supplementary file 1 — Supplementary Material 1. [file 41598_2025_85438_MOESM1_ESM.docx]

**Supplementary material**

[Supplementary figure 1: Directed acyclic graph to identify minimal adjustment sets for the estimation of the total effect of dietary intake of persistent organic pollutants (POPs) on ER-positive breast cancer risk. 3](#_Toc185249863)

[Supplementary figure 2: Heatmap with the Spearman rank correlation coefficients between dietary exposures to 81 POPs in the E3N cohort (N=66,722). 4](#_Toc185249864)

[Supplementary figure 3: Elbow plot representing the dissimilarity of the two clusters being grouped at each step of the algorithm performing variable clustering on dietary exposures to 81 POPs in the E3N cohort (N=66,722). 5](#_Toc185249865)

[Supplementary figure 4: Dendrogram obtained by performing variable clustering on dietary exposures to 81 POPs in the E3N cohort, with the 11 retained clusters framed (N=66,722). 6](#_Toc185249866)

[Supplementary figure 6: Silhouette measures obtained for each POP in the 11 retained clusters identified by performing variable clustering on dietary exposures to 81 POPs in the E3N cohort (N=66,722). 8](#_Toc185249867)

[Supplementary figure 7: Schoenfeld residuals assessing the proportionality of the risks of the exposure variables of the main the Varclus-Cox model in the E3N cohort (N=66,722). 9](#_Toc185249868)

[Supplementary figure 8: Schoenfeld residuals assessing the proportionality of the risks of the exposure variables of the main the PCR-Cox model in the E3N cohort (N=66,722). 10](#_Toc185249869)

[Supplementary figure 9: Schoenfeld residuals assessing the proportionality of the risks of the exposure variables of the main the PLS-Cox model in the E3N cohort (N=20,127). 11](#_Toc185249870)

[Supplementary figure 10: Non-linear association between summary statistics of each cluster of POPs obtained by hierarchical variable clustering and ER-positive breast cancer occurrence in the E3N cohort (N=66,722). Hazard ratios (HR) and 95% Confidence Interval (CI) are estimated by Cox multivariable regression models with penalized cubic spline functions, using the minimum exposure value as reference. 12](#_Toc185249871)

[Supplementary figure 11: Non-linear association between principal components of POPs obtained by PCA and ER-positive breast cancer occurrence in the E3N cohort (N=66,722). Hazard ratios (HR) and 95% Confidence Interval (CI) are estimated by Cox multivariable regression models with penalized cubic spline functions, using the minimum exposure value as reference. 13](#_Toc185249872)

[Supplementary table 1: Description of the clusters obtained by hierarchical variable clustering in the E3N cohort (N=66,722) 14](#_Toc185249873)

[Supplementary table 2: Variance explained and loadings factors for the five principal components obtained by PCA in the E3N cohort (N=66,722) 15](#_Toc185249874)

[Supplementary table 3: Variance explained and loadings factors for the five principal components of POPs obtained by PLS-Cox in relation to ER-positive breast cancer occurrence in the E3N cohort (N=45,595) 17](#_Toc185249875)

[Supplementary table 4: Sensitivity analyses for the association between summary statistics of each cluster of POPs obtained by hierarchical variable clustering and ER-positive breast cancer occurrence in the E3N cohort: additional adjustments on adherence to western and prudent dietary patterns (AS1), on adherence to French dietary guidelines (AS2), and introduction of a 5-year lag between exposure assessment and the start of follow-up (AS3). Hazard ratios (HR) and 95% Confidence Interval (CI) are estimated by Cox multivariable regression models. 19](#_Toc185249876)

[Supplementary table 5: Sensitivity analyses for the associations between principal components of POPs obtained by PCA and ER-positive breast cancer occurrence in the E3N cohort: additional adjustments on adherence to western and prudent dietary patterns (AS1), on adherence to French dietary guidelines (AS2), and introduction of a 5-year lag between exposure assessment and the start of follow-up (AS3). Hazard ratios (HR) and 95% Confidence Interval (CI) are estimated by Cox multivariable regression models. 20](#_Toc185249877)

[Supplementary table 6: Sensitivity analyses for the associations between principal components of POPs obtained by PLS-Cox and ER-positive breast cancer occurrence in the E3N cohort: additional adjustments on adherence to western and prudent dietary patterns (AS1), on adherence to French dietary guidelines (AS2), and introduction of a 5-year lag between exposure assessment and the start of follow-up (AS3). Hazard ratios (HR) and 95% Confidence Interval (CI) are estimated by Cox multivariable regression models. 20](#_Toc185249878)

[Supplementary table 7: Sensitivity analyses for the associations between principal components of POPs obtained by PLS-Cox and ER-positive breast cancer occurrence in the E3N cohort: modeling all continuous adjustment variables with penalized splines (AS4), and fitting the model on the entire original study sample (AS5). Hazard ratios (HR) and 95% Confidence Interval (CI) are estimated by Cox multivariable regression models. 21](#_Toc185249879)

[Supplementary table 8: Reporting checklist for observational studies in nutritional epidemiology based on the STROBE-nut guidelines. 22](#_Toc185249880)

[Annex 1: Formulas of the main Cox models fitted for each of the three approaches (Varclus-Cox, PCR-Cox and PLS-Cox) used to estimate the associations between dietary exposures to combinations of POPs and ER-positive breast cancer risk in the French E3N cohort study. 25](#_Toc185249881)


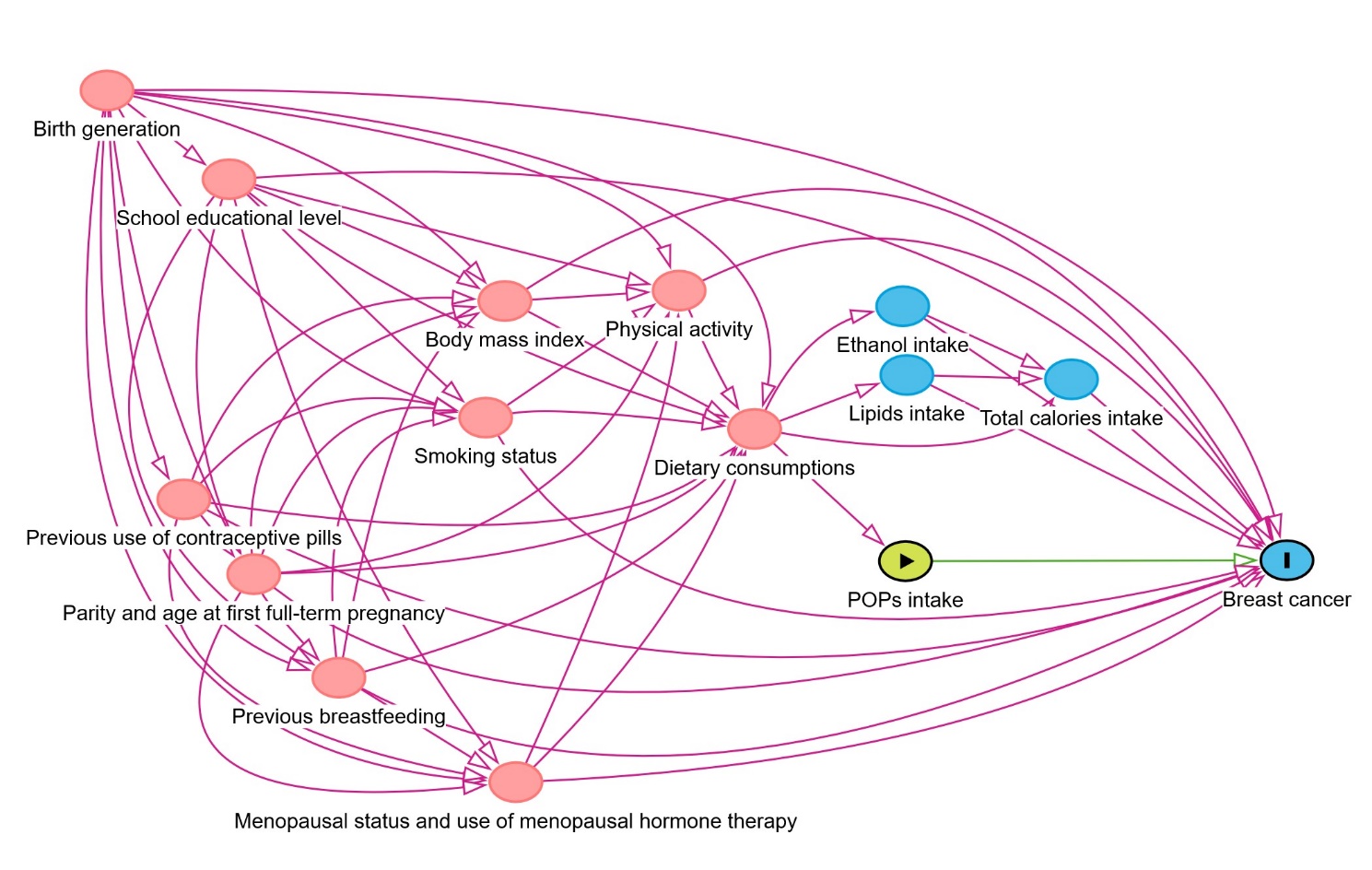


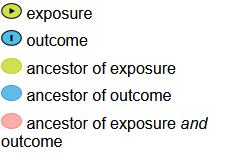

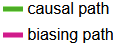


# Supplementary figure 1: Directed acyclic graph to identify minimal adjustment sets for the estimation of the total effect of dietary intake of persistent organic pollutants (POPs) on ER-positive breast cancer risk.

The online software DAGitty was used: <http://www.dagitty.net>

The following minimal sufficient adjustment sets for estimating the total effect of POPs intake on ER-positive breast cancer risk were identified:

1. Birth generation, Body mass index, Ethanol intake, Lipids intake, Menopausal status and use of menopausal hormone therapy, Parity and age at first full-term pregnancy, Physical activity, Previous breastfeeding, Previous use of contraceptive pills, School education level, Smoking status, Total calories intake
2. Dietary consumptions

Since dietary consumption entirely determines dietary intake of POPs (i.e., subjects with exactly the same dietary habits would have exactly the same dietary intake of POPs), it was not possible to precisely adjust for it. The first set of adjustments (1) was therefore used in the main analyses.

If we assume that diet can have a direct effect on breast cancer that is not mediated by lipids, ethanol and total energy intake (the corresponding arrow is not represented in the DAG), this results in residual confounding by diet. Sensitivity analyses were performed by further adjusting the main model on adherence to prudent and western dietary patterns on the one hand, and on adherence to French dietary guidelines on the other.


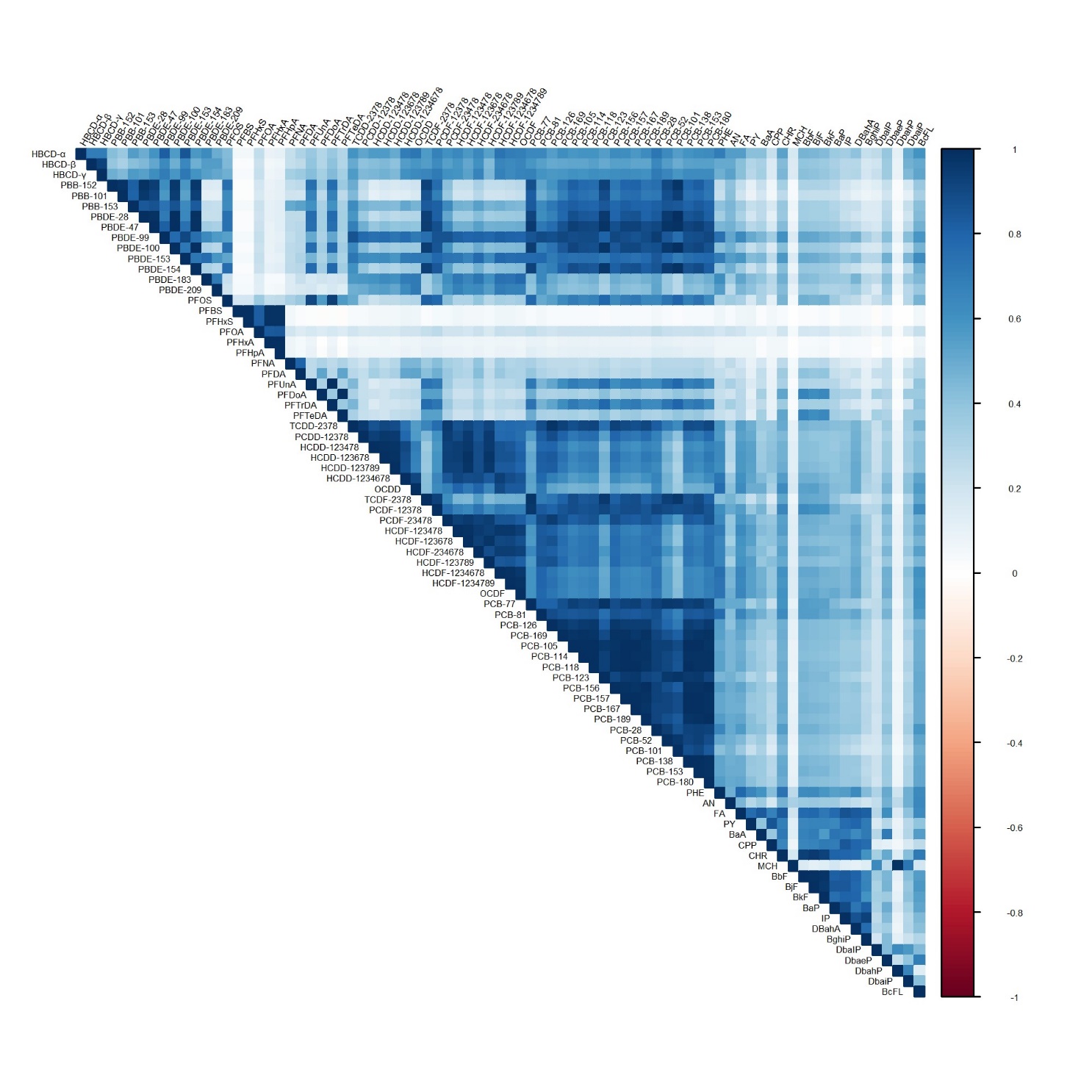


# Supplementary figure 2: Heatmap with the Spearman rank correlation coefficients between dietary exposures to 81 POPs in the E3N cohort (N=66,722).


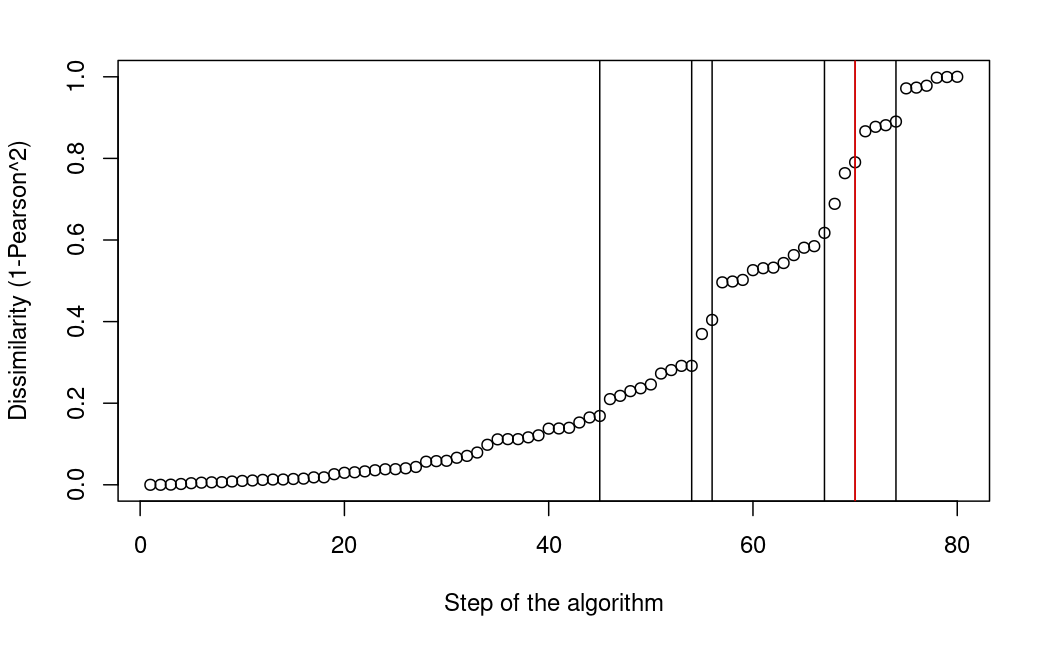


# Supplementary figure 3: Elbow plot representing the dissimilarity of the two clusters being grouped at each step of the algorithm performing variable clustering on dietary exposures to 81 POPs in the E3N cohort (N=66,722).

The vertical lines indicate the candidates elbows for choosing the number of clusters to retain, the red line indicates the elbow chosen.


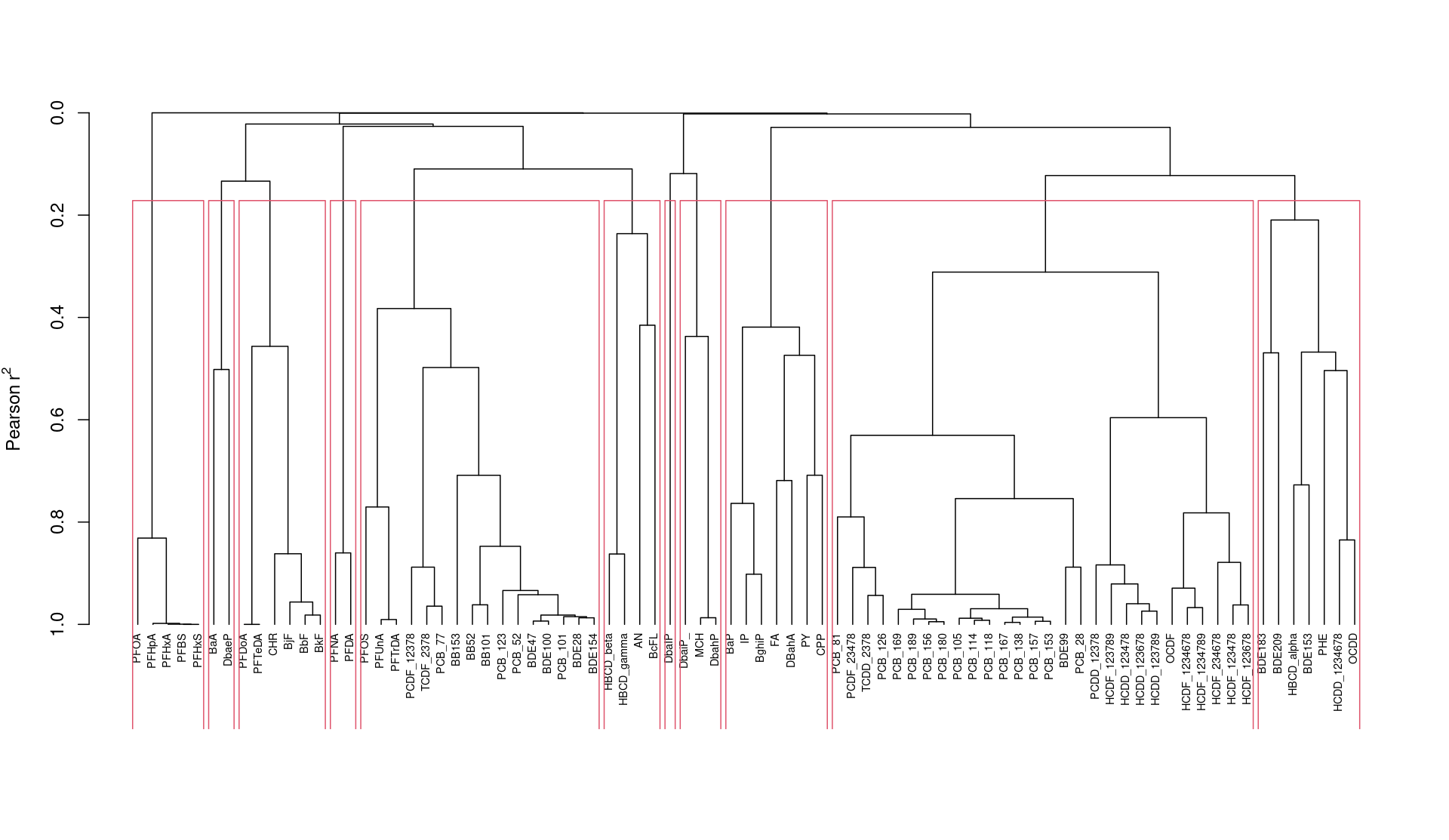


# Supplementary figure 4: Dendrogram obtained by performing variable clustering on dietary exposures to 81 POPs in the E3N cohort, with the 11 retained clusters framed (N=66,722).

**
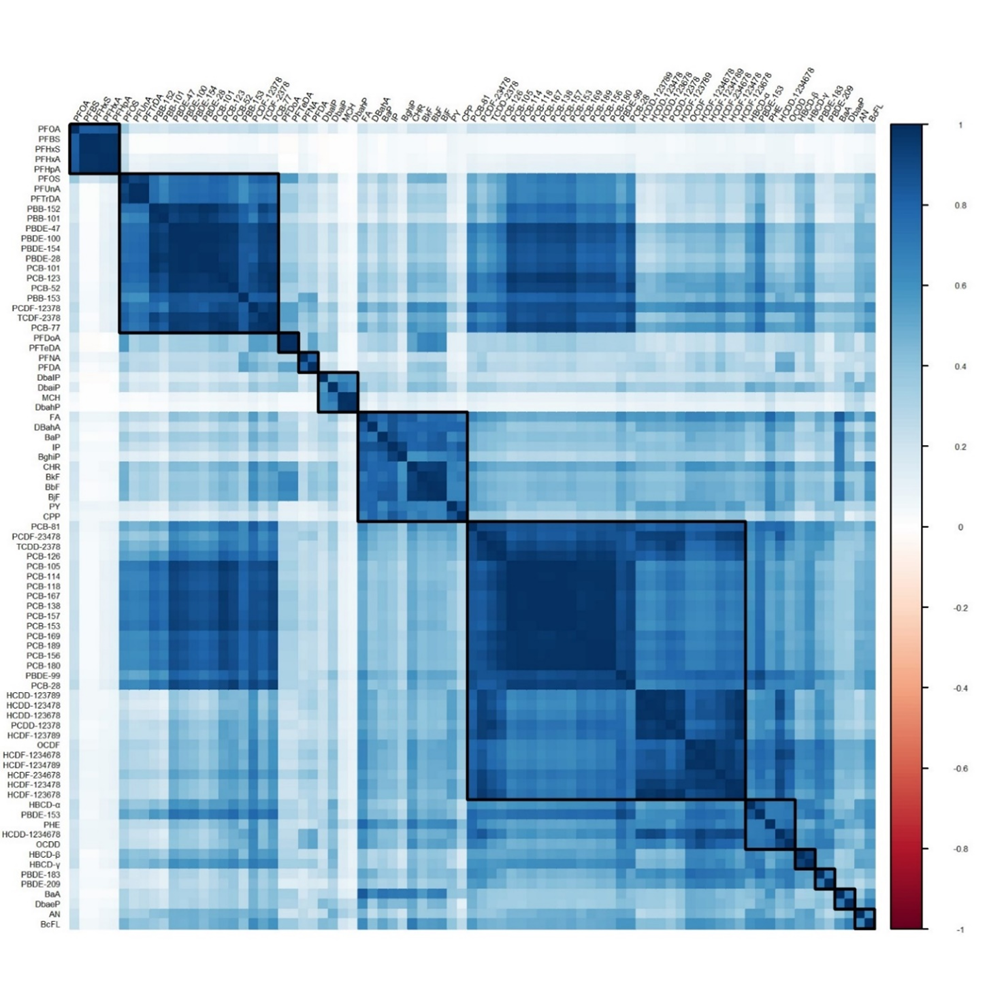
**Supplementary figure 5: Heatmap with the Pearson correlation coefficients between dietary exposures to 81 POPs in the E3N cohort, with the 11 retained clusters framed (N=66,722).


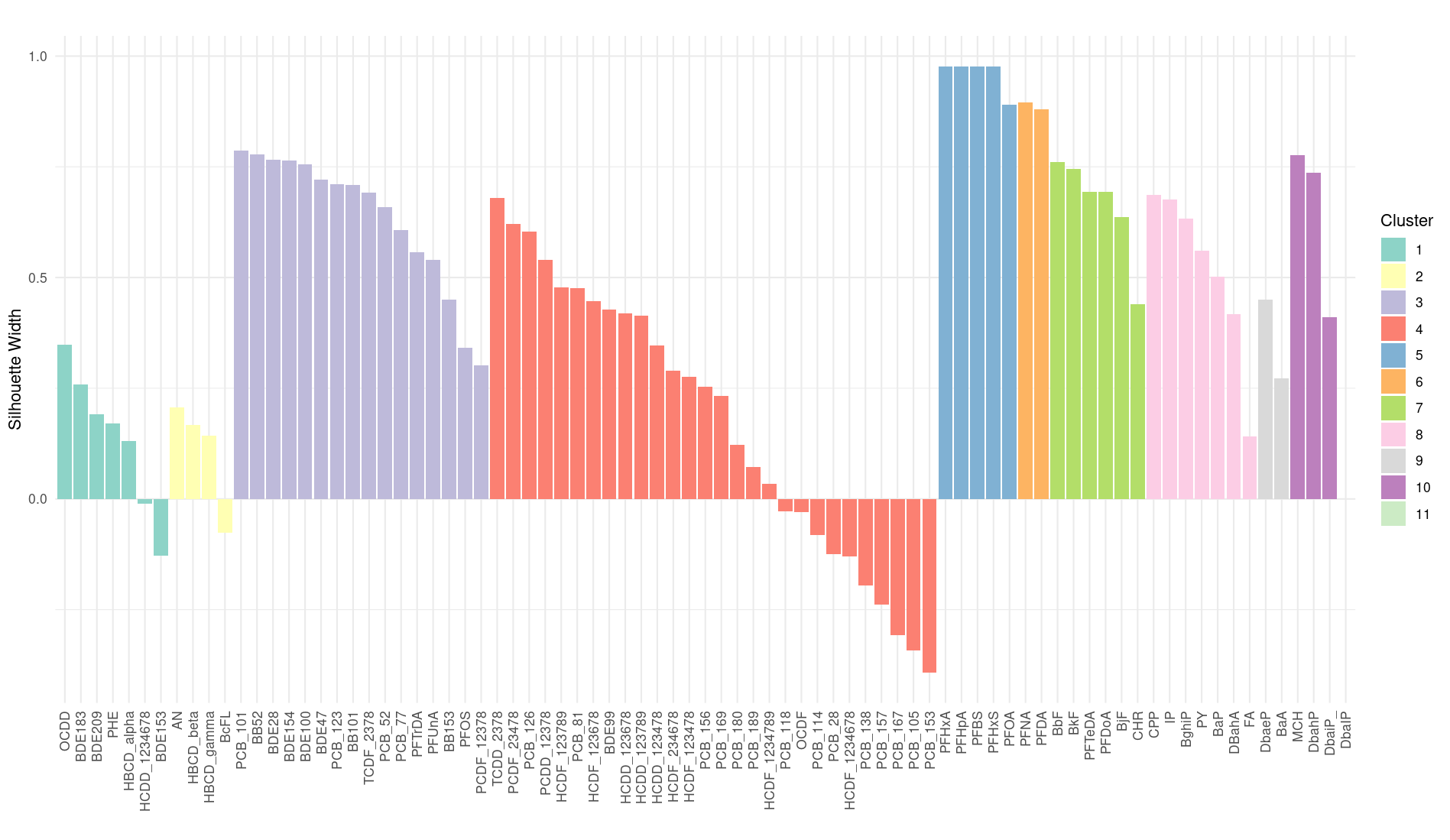


# Supplementary figure 6: Silhouette measures obtained for each POP in the 11 retained clusters identified by performing variable clustering on dietary exposures to 81 POPs in the E3N cohort (N=66,722).


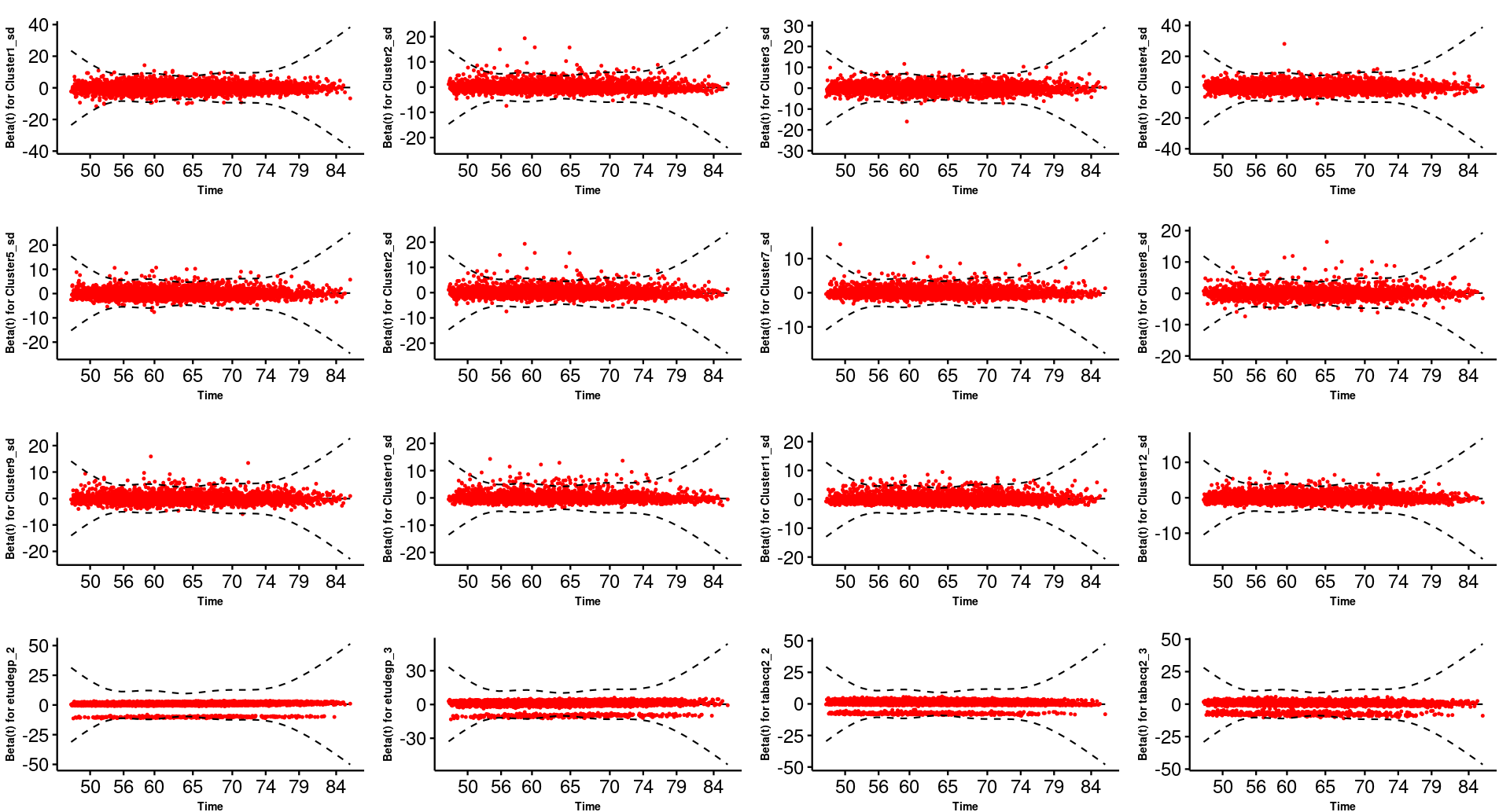


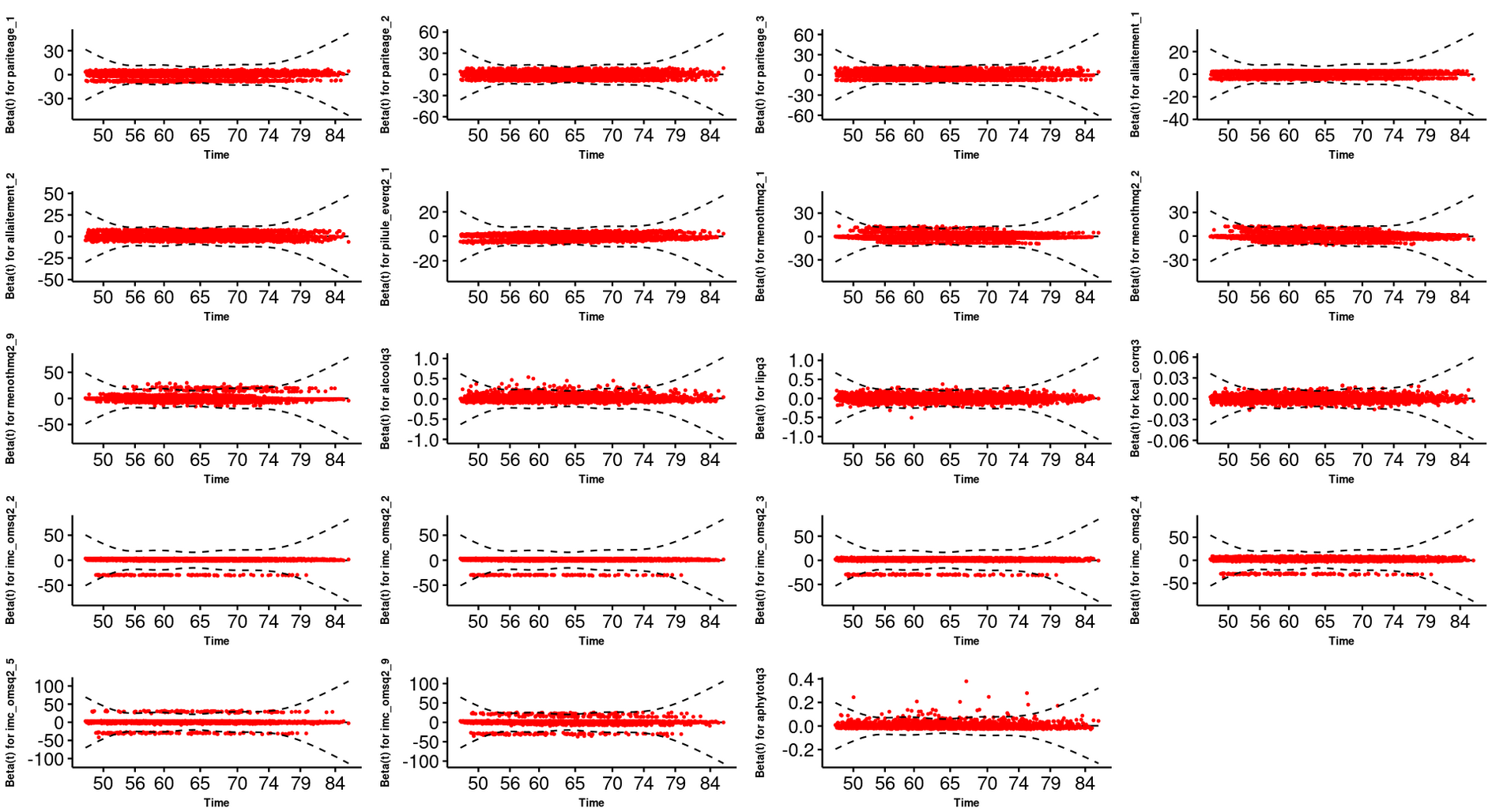


# Supplementary figure 7: Schoenfeld residuals assessing the proportionality of the risks of the exposure variables of the main the Varclus-Cox model in the E3N cohort (N=66,722).


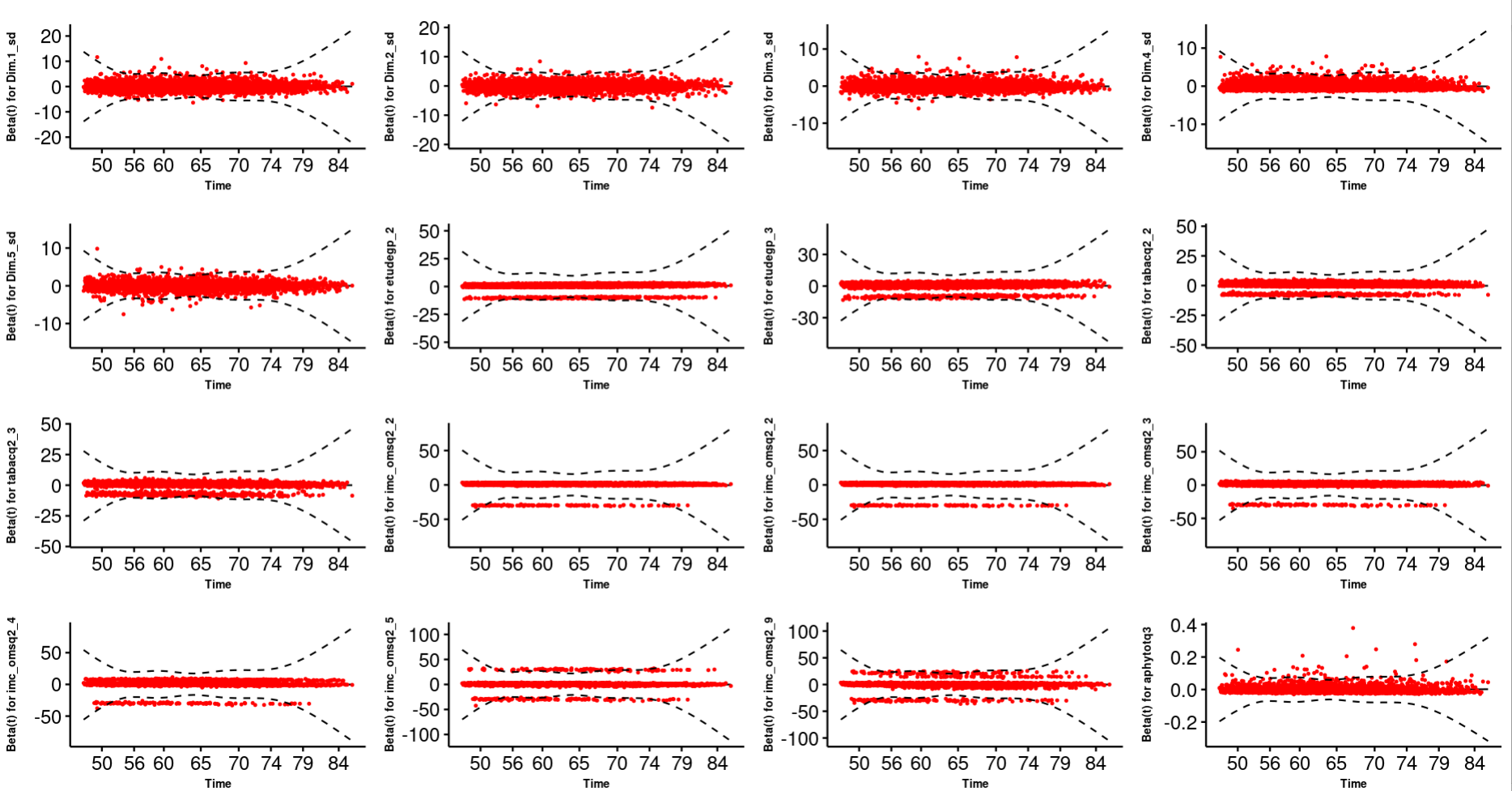


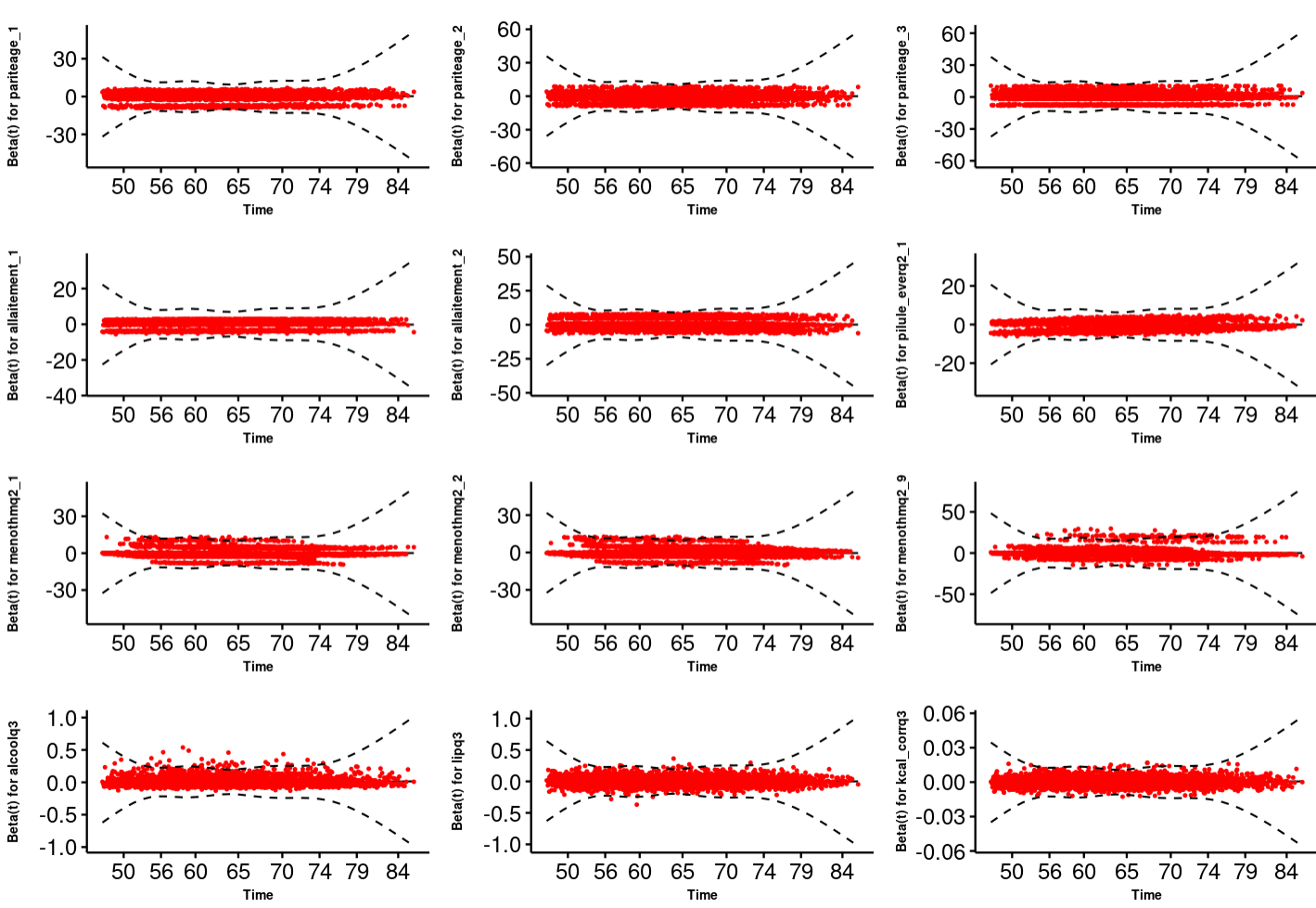


# Supplementary figure 8: Schoenfeld residuals assessing the proportionality of the risks of the exposure variables of the main the PCR-Cox model in the E3N cohort (N=66,722).


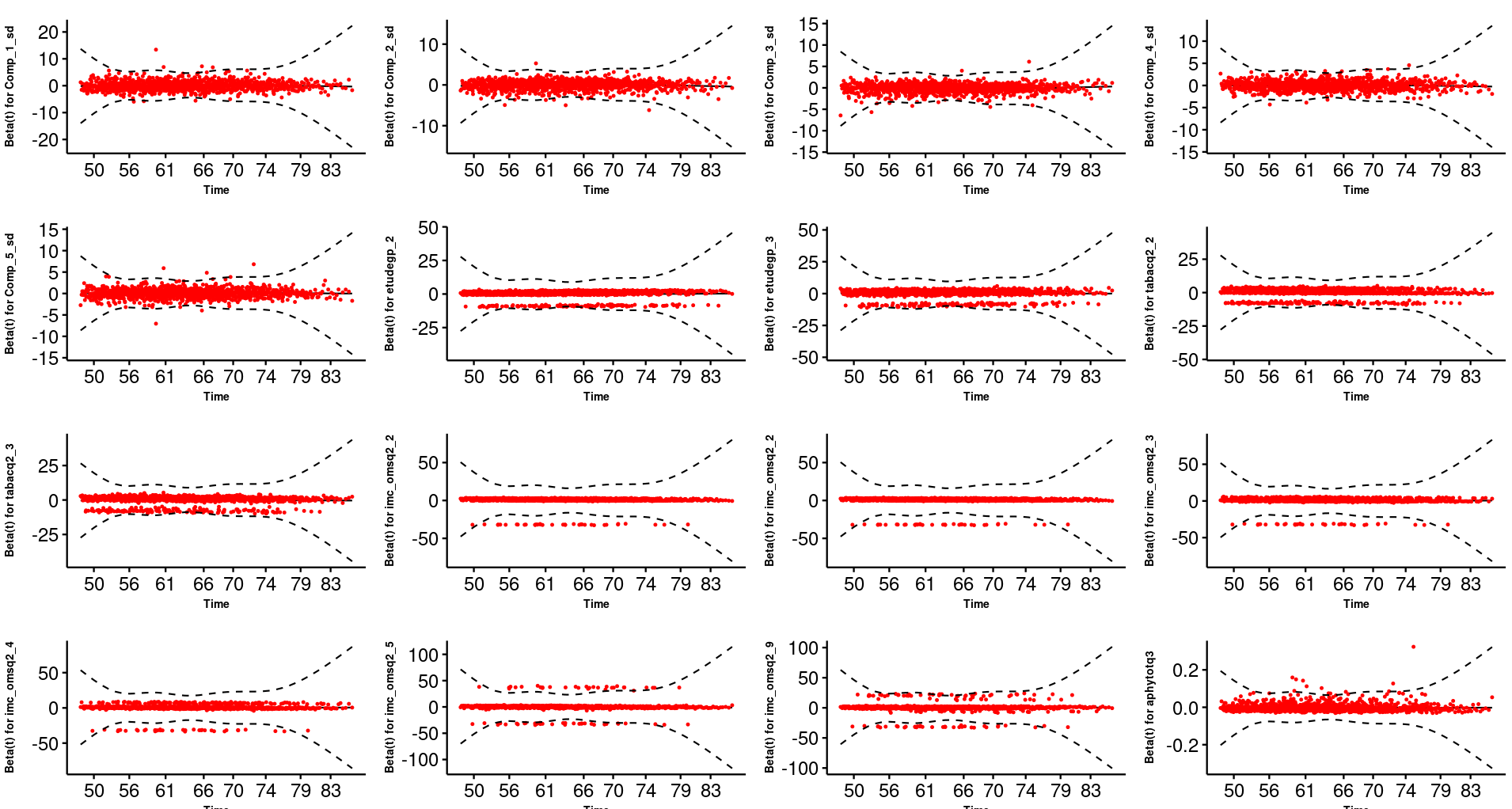


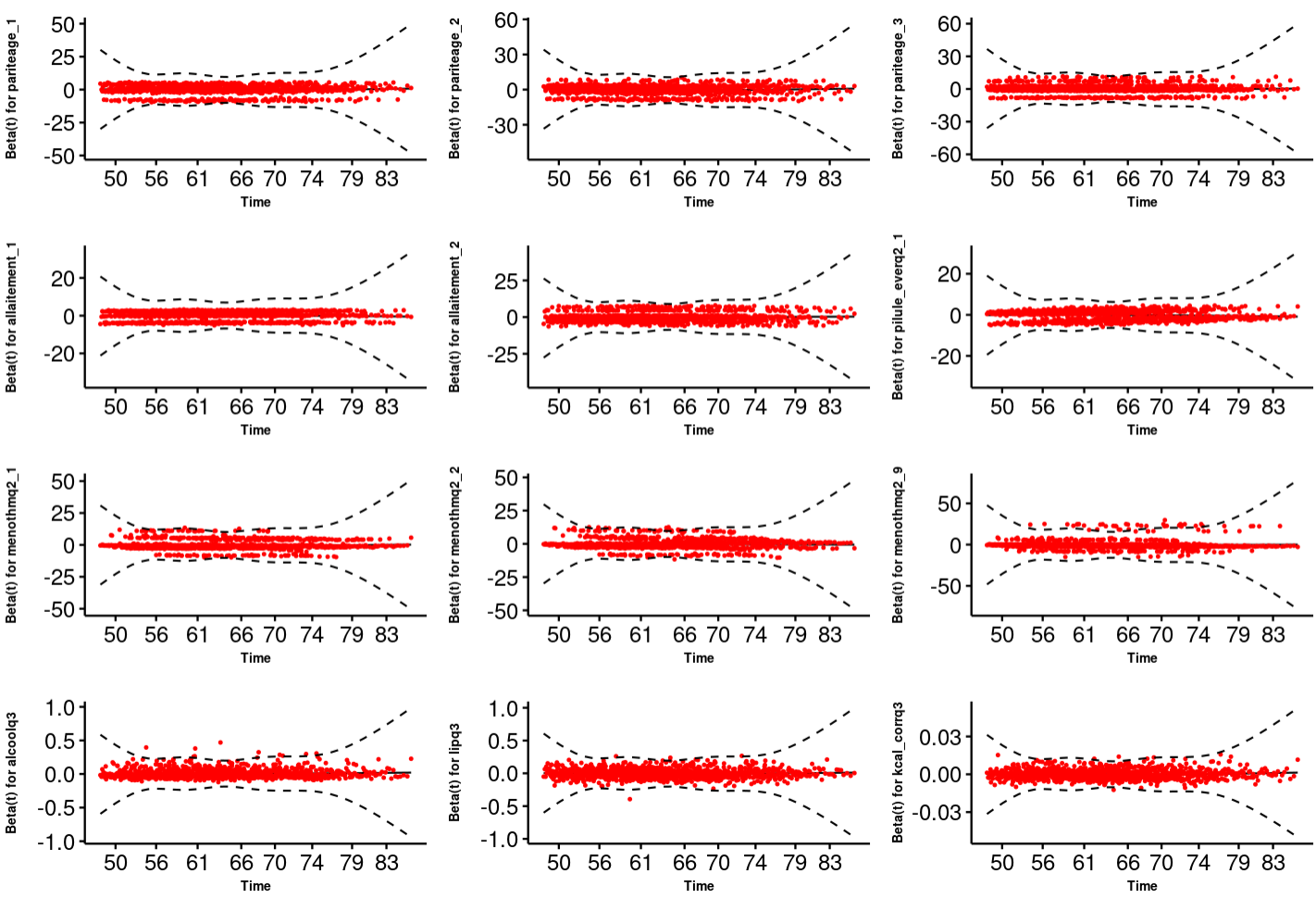


# Supplementary figure 9: Schoenfeld residuals assessing the proportionality of the risks of the exposure variables of the main the PLS-Cox model in the E3N cohort (N=20,127).


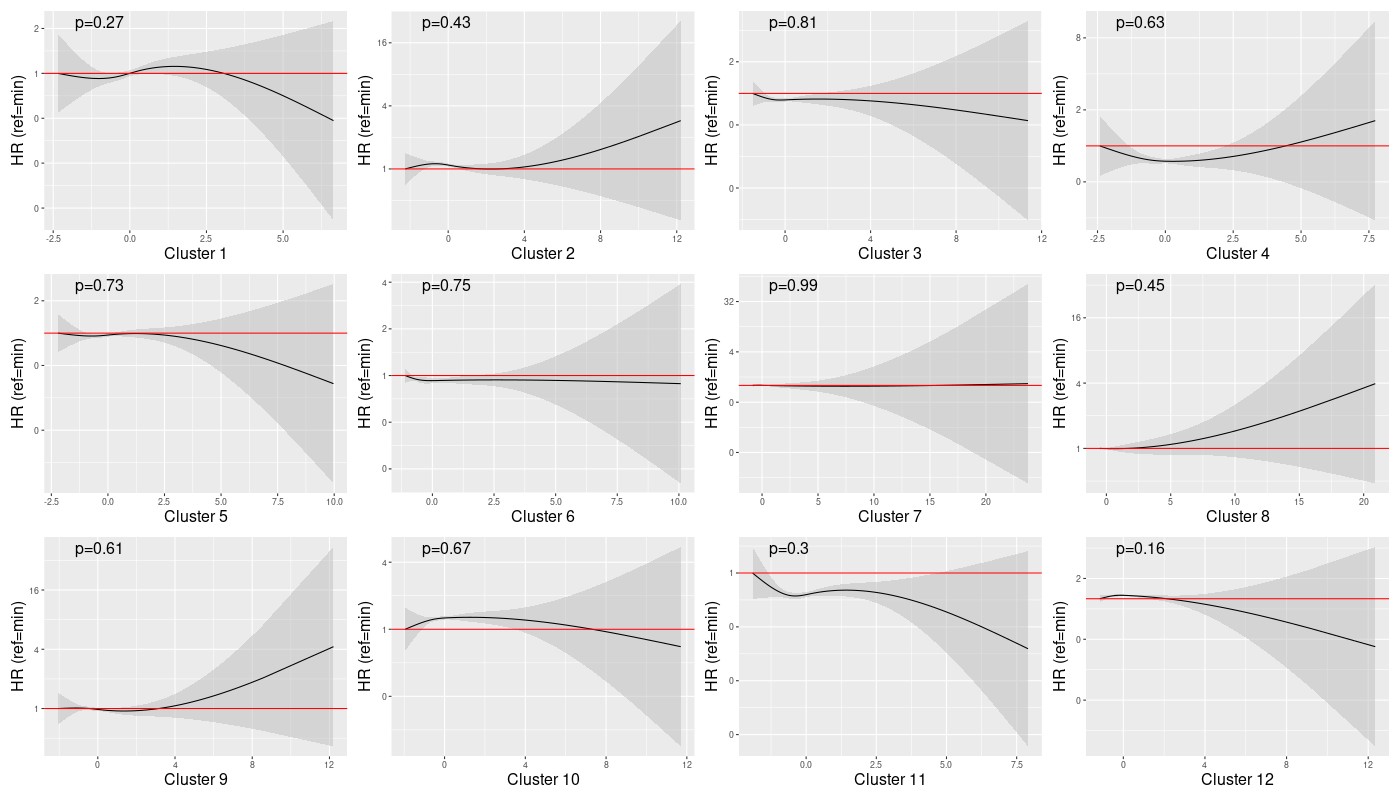


# Supplementary figure 10: Non-linear association between summary statistics of each cluster of POPs obtained by hierarchical variable clustering and ER-positive breast cancer occurrence in the E3N cohort (N=66,722). Hazard ratios (HR) and 95% Confidence Interval (CI) are estimated by Cox multivariable regression models with penalized cubic spline functions, using the minimum exposure value as reference.

p: p-values obtained by log-partial-likelihood ratio test.


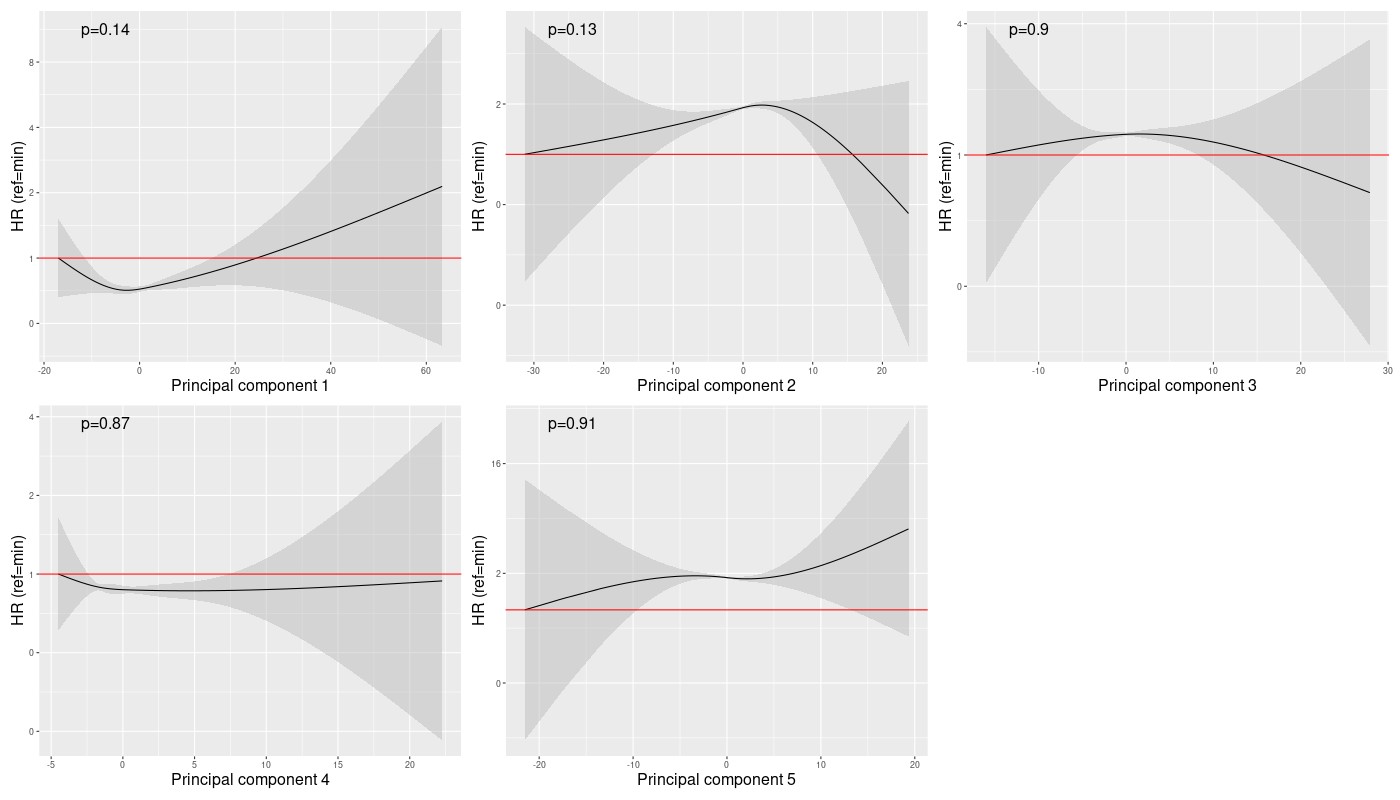


# Supplementary figure 11: Non-linear association between principal components of POPs obtained by PCA and ER-positive breast cancer occurrence in the E3N cohort (N=66,722). Hazard ratios (HR) and 95% Confidence Interval (CI) are estimated by Cox multivariable regression models with penalized cubic spline functions, using the minimum exposure value as reference.

p: p-values obtained by log-partial-likelihood ratio test.

# Supplementary table 1: Description of the clusters obtained by hierarchical variable clustering in the E3N cohort (N=66,722)

| **Cluster n°** | **Number of POPs** | **Range of Pearson correlations between POPs (min** – **max)** | **POPs** |
| --- | --- | --- | --- |
| **1** | N=7 | 0,46 – 0,91 | HBCD-α, PBDE-153, PBDE-183, PBDE-209, HCDD-123478, OCDD, PHE |
| **2** | N=4 | 0,49 – 0,92 | HBCD-β, HBCD-γ, AN, BcFL |
| **3** | N=16 | 0,62 – 1.00 | PBB-52, PBB-101, PBB-153, PBDE-28, PBDE-47, PBDE-100, PBDE-154, PFOS, PFUnA, PFTrDA, TCDF-2378, PCDF-12378, PCB-77, PCB-123, PCB-52, PCB-101 |
| **4** | N=28 | 0,56 – 0.99 | PBDE-99, TCDD-2378, PCDD-12378, HCDD-123478, HCDD-123678, HCDD-123789, PCDF-23478, HCDF-123478, HCDF-123678, HCDF-234678, HCDF-123789, HCDF-1234678, HCDF-1234789, OCDF, PCB-81, PCB-126, PCB-169, PCB-105, PCB-114, PCB-118, PCB-156, PCB-157, PCB-167, PCB-189, PCB-28, PCB-138, PCB-153, PCB-180 |
| **5** | N=5 | 0,91 – 1.00 | PFBS, PFHxS, PFOA, PFHxA, PFHpA |
| **6** | N=2 | 0,93 – 0.93 | PFNA, PFDA |
| **7** | N=6 | 0,68 – 1.00 | PFDoA, PFTeDA, CHR, BbF, BjF, BkF |
| **8** | N=7 | 0.64 – 0.95 | FA, PY, CPP, BaP, IP, DBahA, BghiP |
| **9** | N=2 | 0,71 – 0,71 | BaA, DbaeP |
| **10** | N=3 | 0,66 – 0,99 | MCH, DbahP, DbaiP |
| **11** | N=1 | - | DbaIP |

# Supplementary table 2: Variance explained and loadings factors for the five principal components obtained by PCA in the E3N cohort (N=66,722)

| **Component 1**  51.3% | | **Component 2**  12.5% | | **Component 3**  7.8% | | **Component 4**  6.0% | | **Component 5**  4.4% | |
| --- | --- | --- | --- | --- | --- | --- | --- | --- | --- |
| BDE99 | 0.95 | PY | 0.52 | BjF | 0.67 | PFBS | 0.99 | DbaiP | 0.5 |
| PCB-118 | 0.95 | DBahA | 0.52 | BbF | 0.66 | PFHxS | 0.99 | PFDA | 0.45 |
| PCB-156 | 0.95 | FA | 0.5 | BkF | 0.65 | PFHxA | 0.99 | PFNA | 0.4 |
| PCB-169 | 0.94 | HCDD-123478 | 0.49 | IP | 0.61 | PFHpA | 0.99 | PFDoA | 0.39 |
| PCB-157 | 0.94 | HCDD-1234678 | 0.49 | BaP | 0.6 | PFOA | 0.92 | PFTeDA | 0.39 |
| PCB-167 | 0.94 | HCDD-123789 | 0.48 | CHR | 0.55 | PFOS | 0.15 | MCH | 0.35 |
| PCB-28 | 0.94 | CPP | 0.47 | BghiP | 0.53 | PFNA | 0.09 | HBCD-β | 0.33 |
| PCB-138 | 0.94 | HCDD-123678 | 0.46 | PFDoA | 0.52 | PFDA | 0.05 | HBCD-γ | 0.33 |
| PCB-153 | 0.94 | OCDD | 0.46 | PFTeDA | 0.52 | AN | 0.05 | DbahP | 0.33 |
| PCB-180 | 0.94 | HCDF-123478 | 0.46 | DBahA | 0.47 | HBCD-β | 0.04 | OCDD | 0.32 |
| PCDF-12378 | 0.93 | HCDF-1234678 | 0.46 | CPP | 0.45 | HBCD-γ | 0.04 | DbaIP | 0.32 |
| PCB-126 | 0.93 | BghiP | 0.46 | BaA | 0.43 | OCDD | 0.04 | BcFL | 0.3 |
| PCB-105 | 0.93 | IP | 0.45 | PFOS | 0.36 | BcFL | 0.04 | DbaeP | 0.29 |
| PCB-114 | 0.93 | BDE183 | 0.44 | FA | 0.35 | HBCD-α | 0.03 | AN | 0.24 |
| PCB-189 | 0.93 | HCDF-1234789 | 0.44 | PY | 0.24 | HCDD-1234678 | 0.03 | HBCD-α | 0.23 |
| PCB-77 | 0.92 | HCDF-123678 | 0.43 | TCDF-2378 | 0.22 | HCDF-1234678 | 0.02 | PFOS | 0.19 |
| PCB-81 | 0.92 | OCDF | 0.43 | PFTrDA | 0.21 | DbaeP | 0.02 | HCDD-1234678 | 0.19 |
| TCDD-2378 | 0.9 | PHE | 0.43 | DbaeP | 0.2 | PBB-153 | 0.01 | PCDF-12378 | 0.18 |
| PCDF-23478 | 0.9 | HCDF-123789 | 0.41 | BcFL | 0.2 | PFUnA | 0.01 | BDE183 | 0.14 |
| PCB-123 | 0.88 | HCDF-234678 | 0.36 | DbaIP | 0.18 | PFTrDA | 0.01 | HCDF-1234678 | 0.14 |
| BDE153 | 0.87 | BaA | 0.36 | DbaiP | 0.18 | PCDF-12378 | 0.01 | BDE153 | 0.12 |
| PCB-52 | 0.87 | CHR | 0.36 | PFUnA | 0.16 | HCDF-1234789 | 0.01 | TCDF-2378 | 0.12 |
| TCDF-2378 | 0.85 | BaP | 0.36 | PCDF-12378 | 0.16 | MCH | 0.01 | BDE209 | 0.1 |
| PBDE-47 | 0.84 | BDE209 | 0.35 | PBB-52 | 0.14 | DbahP | 0.01 | HCDF-234678 | 0.1 |
| HCDF-234678 | 0.84 | PCDD-12378 | 0.35 | PCB-77 | 0.14 | DbaiP | 0.01 | OCDF | 0.09 |
| PBDE-100 | 0.82 | BjF | 0.33 | PCB-101 | 0.11 | BDE153 | 0 | BbF | 0.09 |
| PCB-101 | 0.82 | BbF | 0.29 | PFDA | 0.09 | HCDF-234678 | 0 | PCB-77 | 0.08 |
| PBB-153 | 0.8 | BkF | 0.28 | PBB-101 | 0.08 | OCDF | 0 | BkF | 0.08 |
| PBDE-154 | 0.8 | DbaeP | 0.26 | PCB-123 | 0.08 | PCB-28 | 0 | HCDF-1234789 | 0.07 |
| HCDF-123478 | 0.8 | PCDF-23478 | 0.25 | AN | 0.08 | PHE | 0 | PHE | 0.06 |
| HCDF-123678 | 0.79 | DbaiP | 0.22 | PBDE-28 | 0.07 | FA | 0 | CHR | 0.06 |
| HCDF-1234678 | 0.79 | DbaIP | 0.19 | PBDE-154 | 0.07 | PBB-52 | -0.01 | PBB-153 | 0.05 |
| HCDF-1234789 | 0.79 | MCH | 0.16 | MCH | 0.07 | PBB-101 | -0.01 | PFTrDA | 0.05 |
| OCDF | 0.79 | BcFL | 0.16 | DbahP | 0.07 | PBDE-28 | -0.01 | PFUnA | 0.02 |
| PBDE-28 | 0.77 | PCB-81 | 0.15 | PBDE-100 | 0.06 | PBDE-47 | -0.01 | BjF | 0.02 |
| PCDD-12378 | 0.77 | HBCD-α | 0.13 | PFOA | 0.03 | PBDE-100 | -0.01 | PFOA | 0.01 |
| HCDF-123789 | 0.77 | DbahP | 0.13 | PBDE-47 | 0.02 | PBDE-154 | -0.01 | HCDF-123478 | 0 |
| HCDD-123678 | 0.75 | TCDD-2378 | 0.12 | PFNA | 0.01 | BDE209 | -0.01 | BDE99 | -0.02 |
| HBCD-α | 0.73 | PFDA | 0.11 | PCB-52 | 0.01 | PFDoA | -0.01 | PCB-101 | -0.02 |
| HBCD-γ | 0.73 | PFNA | 0.08 | PHE | 0.01 | PFTeDA | -0.01 | PBDE-154 | -0.03 |
| PBB-52 | 0.73 | HBCD-β | 0.07 | PBB-153 | -0.03 | TCDF-2378 | -0.01 | BaA | -0.03 |
| HCDD-123789 | 0.73 | BDE153 | 0.03 | PFBS | -0.03 | HCDF-123478 | -0.01 | PCB-123 | -0.04 |
| HCDD-1234678 | 0.73 | PFBS | 0.02 | PFHxS | -0.03 | PCB-77 | -0.01 | PCB-28 | -0.04 |
| HCDD-123478 | 0.72 | PFHxS | 0.02 | PFHxA | -0.03 | PCB-52 | -0.01 | PCB-52 | -0.04 |
| PFOS | 0.71 | BDE99 | 0.01 | PFHpA | -0.03 | PCB-101 | -0.01 | PBB-52 | -0.05 |
| CHR | 0.69 | PFHxA | 0.01 | PCB-153 | -0.03 | PY | -0.01 | PCB-81 | -0.05 |
| PBB-101 | 0.68 | PFDoA | 0.01 | HBCD-β | -0.04 | BaA | -0.01 | PBDE-100 | -0.06 |
| PHE | 0.68 | PFTeDA | 0.01 | PCB-167 | -0.04 | CHR | -0.01 | HCDD-123478 | -0.06 |
| OCDD | 0.67 | HBCD-γ | 0 | HBCD-γ | -0.06 | BkF | -0.01 | PCDF-23478 | -0.06 |
| FA | 0.67 | PFOA | 0 | HBCD-α | -0.08 | BDE99 | -0.02 | PBDE-28 | -0.07 |
| HBCD-β | 0.65 | PFHpA | -0.01 | PCB-105 | -0.08 | BDE183 | -0.02 | PBDE-47 | -0.07 |
| PFTrDA | 0.65 | PCB-126 | -0.02 | PCB-157 | -0.08 | HCDD-123478 | -0.02 | PFBS | -0.08 |
| BcFL | 0.65 | AN | -0.03 | PCB-138 | -0.08 | PCB-81 | -0.02 | PFHxS | -0.08 |
| PFUnA | 0.63 | PCDF-12378 | -0.16 | BDE153 | -0.11 | PCB-123 | -0.02 | PCB-153 | -0.08 |
| BbF | 0.62 | PCB-169 | -0.19 | PCB-118 | -0.11 | CPP | -0.02 | PFHxA | -0.09 |
| BjF | 0.62 | PCB-156 | -0.19 | PCB-28 | -0.11 | BbF | -0.02 | PFHpA | -0.09 |
| BkF | 0.61 | PCB-28 | -0.2 | PCB-189 | -0.12 | BjF | -0.02 | HCDD-123789 | -0.09 |
| BDE183 | 0.59 | PCB-180 | -0.22 | PCB-114 | -0.13 | IP | -0.02 | HCDF-123678 | -0.09 |
| DBahA | 0.54 | PCB-189 | -0.23 | OCDD | -0.14 | DbaIP | -0.02 | PCB-167 | -0.1 |
| AN | 0.53 | PCB-118 | -0.24 | PCB-169 | -0.14 | HCDD-123678 | -0.03 | PBB-101 | -0.11 |
| PY | 0.51 | PCB-114 | -0.27 | PCB-180 | -0.14 | PCDF-23478 | -0.03 | PCB-157 | -0.11 |
| DbaeP | 0.51 | PCB-138 | -0.27 | PCB-81 | -0.15 | HCDF-123678 | -0.03 | PCB-138 | -0.11 |
| BDE209 | 0.5 | PCB-157 | -0.29 | BDE99 | -0.16 | PCB-105 | -0.03 | HCDD-123678 | -0.12 |
| PFDoA | 0.49 | PCB-167 | -0.3 | PCB-156 | -0.16 | PCB-157 | -0.03 | PCB-118 | -0.13 |
| PFTeDA | 0.49 | PCB-153 | -0.31 | BDE183 | -0.2 | PCB-167 | -0.03 | PCB-105 | -0.14 |
| BaA | 0.48 | PCB-105 | -0.32 | BDE209 | -0.22 | PCB-138 | -0.03 | PCB-189 | -0.14 |
| IP | 0.46 | PCB-77 | -0.33 | PCB-126 | -0.22 | PCB-153 | -0.03 | PCB-180 | -0.14 |
| DbaiP | 0.46 | PFOS | -0.35 | TCDD-2378 | -0.24 | PCB-180 | -0.03 | TCDD-2378 | -0.15 |
| BaP | 0.41 | PBB-153 | -0.38 | OCDF | -0.25 | DBahA | -0.03 | PCB-156 | -0.15 |
| PFDA | 0.4 | TCDF-2378 | -0.42 | PCDF-23478 | -0.27 | BghiP | -0.03 | HCDF-123789 | -0.16 |
| PFNA | 0.37 | PCB-52 | -0.44 | HCDF-1234678 | -0.28 | HCDD-123789 | -0.04 | PCB-169 | -0.16 |
| BghiP | 0.35 | PCB-123 | -0.45 | HCDD-1234678 | -0.29 | HCDF-123789 | -0.04 | PCB-114 | -0.17 |
| CPP | 0.34 | PFTrDA | -0.47 | HCDF-234678 | -0.29 | PCB-169 | -0.04 | FA | -0.17 |
| DbaIP | 0.3 | PFUnA | -0.48 | HCDF-1234789 | -0.3 | PCB-114 | -0.04 | PCB-126 | -0.18 |
| PFOA | 0.25 | PBDE-47 | -0.51 | PCDD-12378 | -0.35 | PCB-118 | -0.04 | PCDD-12378 | -0.19 |
| MCH | 0.16 | PCB-101 | -0.54 | HCDD-123789 | -0.35 | PCB-156 | -0.04 | DBahA | -0.25 |
| DbahP | 0.12 | PBDE-100 | -0.55 | HCDF-123478 | -0.35 | PCB-189 | -0.04 | BaP | -0.37 |
| PFHpA | 0.09 | PBDE-154 | -0.56 | HCDF-123789 | -0.37 | BaP | -0.04 | PY | -0.4 |
| PFHxA | 0.07 | PBDE-28 | -0.6 | HCDD-123478 | -0.38 | TCDD-2378 | -0.05 | IP | -0.4 |
| PFBS | 0.06 | PBB-52 | -0.65 | HCDF-123678 | -0.38 | PCB-126 | -0.05 | BghiP | -0.48 |
| PFHxS | 0.06 | PBB-101 | -0.67 | HCDD-123678 | -0.39 | PCDD-12378 | -0.06 | CPP | -0.56 |

# Supplementary table 3: Variance explained and loadings factors for the five principal components of POPs obtained by PLS-Cox in relation to ER-positive breast cancer occurrence in the E3N cohort (N=45,595)

| **Component 1**  50.3% | | **Component 2**  11.9% | | **Component 3**  6.2% | | **Component 4**  5.8% | | **Component 5**  3.0% | |
| --- | --- | --- | --- | --- | --- | --- | --- | --- | --- |
| HCDF-1234789 | 0,18 | HCDF-1234789 | 0,22 | PCB-157 | 0,27 | PCB-157 | 0,59 | HCDF-1234789 | 0,37 |
| DBahA | 0,18 | DBahA | 0,22 | PCB-153 | 0,27 | HCDF-1234789 | 0,37 | HCDF-1234678 | 0,32 |
| OCDF | 0,17 | OCDF | 0,20 | PCB-105 | 0,22 | PCB-105 | 0,37 | OCDF | 0,27 |
| HCDF-1234678 | 0,16 | HCDF-1234678 | 0,18 | HCDF-1234789 | 0,13 | HCDF-1234678 | 0,31 | OCDD | 0,17 |
| CHR | 0,16 | CHR | 0,18 | DBahA | 0,12 | OCDF | 0,29 | PCB-101 | 0,15 |
| OCDD | 0,15 | OCDD | 0,15 | OCDF | 0,10 | PCB-118 | 0,26 | DBahA | 0,15 |
| HCDF-123478 | 0,15 | FA | 0,14 | PCB-118 | 0,09 | PCB-153 | 0,26 | PCB-157 | 0,14 |
| FA | 0,15 | BbF | 0,14 | PCB-167 | 0,09 | PCB-114 | 0,23 | PCB-52 | 0,12 |
| HCDD-123789 | 0,14 | BkF | 0,14 | PCB-138 | 0,09 | PCB-180 | 0,23 | DbaiP- | 0,12 |
| HCDD-1234678 | 0,14 | HCDF-123478 | 0,13 | PFTrDA | 0,07 | HCDF-123478 | 0,20 | HBCD-α | 0,11 |
| PCDF-12378 | 0,14 | BjF | 0,13 | HCDF-1234678 | 0,07 | PCB-156 | 0,20 | PBB-52 | 0,11 |
| HCDF-123678 | 0,14 | HCDD-1234678 | 0,11 | PCB-114 | 0,07 | OCDD | 0,14 | PBB-153 | 0,11 |
| HCDF-234678 | 0,14 | PY | 0,11 | CHR | 0,07 | PCB-169 | 0,13 | HCDD-1234678 | 0,11 |
| BbF | 0,14 | BaA | 0,11 | PFUnA | 0,06 | DBahA | 0,13 | PBDE-100 | 0,10 |
| BjF | 0,14 | BcFL | 0,11 | PFOS | 0,05 | HCDD-1234678 | 0,10 | PBDE-154 | 0,10 |
| BkF | 0,14 | PFDoA | 0,10 | OCDD | 0,05 | PCB-138 | 0,10 | BDE153 | 0,09 |
| HBCD-α | 0,13 | PFTeDA | 0,10 | PFDoA | 0,04 | HBCD-α | 0,08 | PFOS | 0,08 |
| BDE99 | 0,13 | HBCD-α | 0,09 | PFTeDA | 0,04 | PBB-153 | 0,08 | PFTrDA | 0,08 |
| BDE153 | 0,13 | HCDD-123478 | 0,09 | BkF | 0,04 | PFUnA | 0,08 | CHR | 0,08 |
| TCDD-2378 | 0,13 | HCDD-123678 | 0,09 | DbaiP- | 0,04 | PFTrDA | 0,08 | PFBS | 0,07 |
| HCDD-123478 | 0,13 | HCDD-123789 | 0,09 | HBCD-α | 0,03 | PCB-189 | 0,08 | PFHxS | 0,07 |
| HCDD-123678 | 0,13 | DbaiP- | 0,09 | PBDE-100 | 0,03 | DbaiP- | 0,07 | PFHxA | 0,07 |
| PCDF-23478 | 0,13 | PFOS | 0,08 | HCDF-123478 | 0,03 | HCDD-123678 | 0,06 | PFHpA | 0,07 |
| BcFL | 0,13 | PFTrDA | 0,08 | PCB-180 | 0,03 | HCDF-123678 | 0,05 | PFUnA | 0,07 |
| PFOS | 0,12 | HCDF-123678 | 0,08 | BaA | 0,03 | BDE153 | 0,04 | PBDE-47 | 0,06 |
| PCDD-12378 | 0,12 | CPP | 0,08 | BbF | 0,03 | PFOS | 0,04 | PCB-123 | 0,06 |
| HCDF-123789 | 0,12 | DbaIP | 0,08 | DbaIP | 0,03 | HCDD-123478 | 0,04 | PCDF-12378 | 0,05 |
| PCB-126 | 0,12 | BDE183 | 0,07 | BcFL | 0,03 | HCDD-123789 | 0,04 | BcFL | 0,05 |
| PCB-169 | 0,12 | PFUnA | 0,07 | PBB-153 | 0,02 | DbaIP | 0,03 | PBDE-28 | 0,04 |
| PCB-156 | 0,12 | PHE | 0,07 | PBDE-154 | 0,02 | BcFL | 0,02 | PCB-105 | 0,04 |
| PHE | 0,12 | PFDA | 0,06 | PCB-123 | 0,02 | BDE183 | -0,01 | DbaIP | 0,04 |
| PY | 0,12 | HCDF-234678 | 0,05 | FA | 0,02 | PFBS | -0,01 | FA | 0,03 |
| BaA | 0,12 | IP | 0,05 | PY | 0,02 | PFHxS | -0,01 | BDE183 | 0,02 |
| BDE183 | 0,11 | BDE153 | 0,04 | BjF | 0,02 | PFHxA | -0,01 | PFDoA | 0,02 |
| PFDoA | 0,11 | PCDD-12378 | 0,04 | PBDE-47 | 0,01 | PFHpA | -0,01 | PFTeDA | 0,02 |
| PFTrDA | 0,11 | HCDF-123789 | 0,04 | PFDA | 0,01 | PFDoA | -0,01 | HCDF-123478 | 0,02 |
| PFTeDA | 0,11 | DbaeP | 0,04 | PCB-156 | 0,01 | PFTeDA | -0,01 | PHE | 0,02 |
| PCB-77 | 0,11 | BaP | 0,03 | CPP | 0,01 | PCB-52 | -0,01 | PY | 0,02 |
| PCB-118 | 0,11 | BghiP | 0,03 | PBB-52 | 0,00 | PY | -0,01 | BDE99 | 0,01 |
| PCB-157 | 0,11 | PFNA | 0,02 | PBDE-28 | 0,00 | PFDA | -0,02 | BaA | 0,01 |
| PCB-167 | 0,11 | PCDF-12378 | 0,02 | HCDD-123789 | 0,00 | BaA | -0,02 | BkF | 0,01 |
| PCB-189 | 0,11 | BDE209 | 0,01 | HCDD-1234678 | 0,00 | PBDE-154 | -0,03 | PFDA | 0,00 |
| PCB-138 | 0,11 | PBB-153 | 0,00 | PCB-169 | 0,00 | CHR | -0,03 | PCB-28 | 0,00 |
| PCB-153 | 0,11 | AN | -0,01 | PCB-189 | 0,00 | PFNA | -0,04 | PCB-180 | 0,00 |
| PCB-180 | 0,11 | TCDD-2378 | -0,03 | PCB-101 | 0,00 | PCDD-12378 | -0,04 | HCDD-123789 | -0,01 |
| PBB-153 | 0,10 | PCDF-23478 | -0,05 | PBB-101 | -0,01 | HCDF-234678 | -0,04 | BbF | -0,01 |
| PFUnA | 0,10 | PBB-52 | -0,06 | BDE183 | -0,01 | PHE | -0,04 | PBB-101 | -0,02 |
| PCB-81 | 0,10 | PBDE-154 | -0,06 | PFNA | -0,01 | FA | -0,04 | PFNA | -0,02 |
| PCB-105 | 0,10 | TCDF-2378 | -0,06 | HCDD-123478 | -0,01 | CPP | -0,05 | BjF | -0,02 |
| PCB-114 | 0,10 | PCB-101 | -0,07 | HCDD-123678 | -0,01 | PCDF-12378 | -0,06 | HCDD-123678 | -0,03 |
| PCB-28 | 0,10 | HBCD-β | -0,08 | PCDF-12378 | -0,01 | HCDF-123789 | -0,06 | PCB-169 | -0,03 |
| DbaiP- | 0,10 | PBB-101 | -0,08 | BDE153 | -0,02 | PCB-167 | -0,06 | PCB-118 | -0,03 |
| TCDF-2378 | 0,09 | PBDE-28 | -0,08 | HCDF-123678 | -0,02 | AN | -0,07 | PCB-153 | -0,03 |
| PCB-123 | 0,09 | PBDE-100 | -0,08 | PCB-52 | -0,02 | BkF | -0,07 | AN | -0,03 |
| CPP | 0,09 | PCB-123 | -0,08 | DbaeP | -0,02 | DbaeP | -0,07 | CPP | -0,03 |
| IP | 0,09 | PBDE-47 | -0,09 | PHE | -0,03 | PBDE-47 | -0,08 | DbaeP | -0,03 |
| DbaeP | 0,09 | PCB-126 | -0,09 | PCDD-12378 | -0,04 | BDE209 | -0,08 | PCB-156 | -0,04 |
| PFDA | 0,08 | PCB-52 | -0,09 | AN | -0,04 | PBDE-100 | -0,09 | BDE209 | -0,05 |
| PCB-52 | 0,08 | MCH | -0,09 | BaP | -0,04 | TCDD-2378 | -0,09 | PFOA | -0,05 |
| DbaIP | 0,08 | PCB-77 | -0,10 | IP | -0,04 | PCB-126 | -0,10 | HCDD-123478 | -0,05 |
| PBDE-47 | 0,07 | PCB-169 | -0,10 | BghiP | -0,04 | BbF | -0,10 | PCB-114 | -0,05 |
| PBDE-100 | 0,07 | PCB-153 | -0,10 | BDE209 | -0,05 | BDE99 | -0,11 | PCB-189 | -0,05 |
| PBDE-154 | 0,07 | PCB-157 | -0,11 | HCDF-234678 | -0,06 | PBB-52 | -0,13 | PCDD-12378 | -0,07 |
| BDE209 | 0,07 | PCB-167 | -0,11 | HCDF-123789 | -0,06 | PBB-101 | -0,13 | TCDF-2378 | -0,07 |
| PCB-101 | 0,07 | PCB-189 | -0,11 | TCDF-2378 | -0,07 | PBDE-28 | -0,13 | HCDF-234678 | -0,08 |
| BaP | 0,07 | PCB-180 | -0,11 | PFBS | -0,09 | PCB-101 | -0,13 | PCB-77 | -0,09 |
| PBDE-28 | 0,06 | DbahP | -0,11 | PFHxS | -0,09 | BjF | -0,13 | MCH | -0,09 |
| PFNA | 0,06 | PCB-156 | -0,12 | PFHxA | -0,09 | PFOA | -0,14 | TCDD-2378 | -0,11 |
| AN | 0,06 | BDE99 | -0,13 | TCDD-2378 | -0,09 | MCH | -0,14 | HCDF-123678 | -0,11 |
| BghiP | 0,06 | PFBS | -0,13 | MCH | -0,09 | PCB-28 | -0,15 | HCDF-123789 | -0,11 |
| HBCD-β | 0,05 | PFHxS | -0,13 | PFHpA | -0,10 | BaP | -0,16 | BaP | -0,11 |
| PBB-52 | 0,05 | PFHxA | -0,13 | PCB-77 | -0,10 | BghiP | -0,16 | BghiP | -0,11 |
| HBCD-γ | 0,04 | PFHpA | -0,13 | DbahP | -0,10 | DbahP | -0,16 | DbahP | -0,11 |
| PBB-101 | 0,03 | PCB-105 | -0,13 | PCB-126 | -0,11 | PCDF-23478 | -0,17 | IP | -0,13 |
| MCH | -0,04 | PCB-138 | -0,13 | HBCD-β | -0,14 | IP | -0,18 | HBCD-β | -0,14 |
| DbahP | -0,05 | PCB-114 | -0,14 | PFOA | -0,14 | HBCD-β | -0,23 | PCB-126 | -0,14 |
| PFOA | -0,07 | PCB-118 | -0,14 | PCDF-23478 | -0,15 | TCDF-2378 | -0,23 | PCB-138 | -0,16 |
| PFBS | -0,08 | HBCD-γ | -0,15 | BDE99 | -0,18 | PCB-77 | -0,31 | PCDF-23478 | -0,20 |
| PFHxS | -0,08 | PFOA | -0,15 | PCB-28 | -0,19 | HBCD-γ | -0,36 | HBCD-γ | -0,24 |
| PFHxA | -0,08 | PCB-28 | -0,18 | HBCD-γ | -0,21 | PCB-123 | -0,36 | PCB-167 | -0,25 |
| PFHpA | -0,08 | PCB-81 | -0,29 | PCB-81 | -0,43 | PCB-81 | -0,72 | PCB-81 | -0,53 |

# Supplementary table 4: Sensitivity analyses for the association between summary statistics of each cluster of POPs obtained by hierarchical variable clustering and ER-positive breast cancer occurrence in the E3N cohort: additional adjustments on adherence to western and prudent dietary patterns (AS1), on adherence to French dietary guidelines (AS2), and introduction of a 5-year lag between exposure assessment and the start of follow-up (AS3). Hazard ratios (HR) and 95% Confidence Interval (CI) are estimated by Cox multivariable regression models.

|  | AS1 (N=66,722) | | AS2 (N=66,722) | | AS3 (N=63,456) | | AS4 (N=66,722) | |
| --- | --- | --- | --- | --- | --- | --- | --- | --- |
|  | HR [95% CI] | p-value | HR [95% CI] | p-value | HR [95% CI] | p-value | HR [95% CI] | p-value |
| Cluster 1 summary statistic, for 1 SD increase | 1.00 [0.92 - 1.09] | 0.998 | 1.03 [0.96 - 1.10] | 0.483 | 1.04 [0.96 - 1.12] | 0.388 | 1.05 [0.98 - 1.13] | 0.137 |
| Cluster 2 summary statistic, for 1 SD increase | 0.98 [0.92 - 1.04] | 0.526 | 0.96 [0.91 - 1.02] | 0.177 | 0.97 [0.91 - 1.03] | 0.286 | 0.97 [0.91 - 1.02] | 0.224 |
| Cluster 3 summary statistic, for 1 SD increase | 0.99 [0.93 - 1.07] | 0.851 | 1.00 [0.94 - 1.07] | 1.00 | 1.00 [0.94- 1.08] | 0.925 | 1.00 [0.93 - 1.06] | 0.893 |
| Cluster 4 summary statistic, for 1 SD increase | 1.03 [0.93 - 1.14] | 0.603 | 1.00 [0.92 - 1.09] | 0.943 | 1.00 [0.91- 1.10] | 0.972 | 1.00 [0.91 - 1.09] | 0.930 |
| Cluster 5 summary statistic, for 1 SD increase | 0.99 [0.96 - 1.02] | 0.595 | 0.99 [0.96 - 1.02] | 0.532 | 0.98 [0.95 - 1.02] | 0.379 | 0.99 [0.96 - 1.02] | 0.521 |
| Cluster 6 summary statistic, for 1 SD increase | 1.00 [0.96 - 1.03] | 0.858 | 0.99 [0.96 - 1.03] | 0.743 | 0.99 [0.95 - 1.03] | 0.647 | 0.99 [0.96 - 1.03] | 0.733 |
| Cluster 7 summary statistic, for 1 SD increase | 1.02 [0.97 - 1.07] | 0.493 | 1.02 [0.97 - 1.07] | 0.402 | 1.04 [0.98 - 1.09] | 0.199 | 1.02 [0.97 - 1.07] | 0.357 |
| Cluster 8 summary statistic, for 1 SD increase | 1.01 [0.95 - 1.06] | 0.845 | 1.00 [0.95 - 1.05] | 0.936 | 1.00 [0.94 - 1.05] | 0.938 | 1.00 [0.96 - 1.05] | 0.870 |
| Cluster 9 summary statistic, for 1 SD increase | 1.01 [0.96 - 1.05] | 0.820 | 1.01 [0.96 - 1.06] | 0.666 | 1.00 [0.95 - 1.05] | 0.917 | 1.00 [0.96 - 1.05] | 0.983 |
| Cluster 10 summary statistic, for 1 SD increase | 0.98 [0.94 - 1.01] | 0.195 | 0.98 [0.94 - 1.01] | 0.231 | 0.97 [0.93 - 1.01] | 0.158 | 1.98 [0.95 - 1.02] | 0.368 |
| Cluster 11 summary statistic, for 1 SD increase | 1.00 [0.97 - 1.04] | 0.814 | 1.01 [0.98 - 1.05] | 0.519 | 1.01 [0.97 - 1.05] | 0.581 | 1.01 [0.98 - 1.05] | 0.436 |

SD: standard deviation.

Use of age as the time-scale (years), stratification of the baseline hazard on birth generation (≤1930; (1930-1935]; (1935-1940]; (1940;1945]; >1945), adjustment on school education level (<12 years; 12 to 14 years; >14 years), smoking status (non-smoker; former smoker; current smoker), body mass index (<18.5; [18.5-22.5); [22.5-25); [25-30); ≥30 kg/m2; missing values), parity and age at FFTP (nulliparous; one or two children and age at FFTP<30; more than two children and age at FFTP<30; age at FFTP≥30), cumulated duration of previous breastfeeding (no breastfeeding: less than 6 months of breastfeeding; at least 6 months of breastfeeding), utilization of contraceptive pill (ever; never), menopausal status and recent use of MHT (pre-menopaused, menopaused with recent use oh MHT; menopaused without recent use of MHT, menopaused and missing data on recent used of MHT), physical activity (continuously in metabolic equivalents of task-hour/week), daily alcohol intake (continuously in g of ethanol/day), daily lipids intake (continuously in g/day), daily total energy intake except from alcohol and lipid (continuously in kcal/day), and mutual adjustment of each cluster summary statistic to each other.

AS1: Supplementary adjusted on adherence to western dietary pattern (continuously) and adherence to prudent dietary patterns (continuously).

AS2: Supplementary adjusted on adherence to French dietary guidelines (continuously).

AS3: Introduction of a 5-year lag between exposure assessment and the start of follow-up.

AS4: Use of a reduced set of adjustment, excluding the grey covariates.

# Supplementary table 5: Sensitivity analyses for the associations between principal components of POPs obtained by PCA and ER-positive breast cancer occurrence in the E3N cohort: additional adjustments on adherence to western and prudent dietary patterns (AS1), on adherence to French dietary guidelines (AS2), and introduction of a 5-year lag between exposure assessment and the start of follow-up (AS3). Hazard ratios (HR) and 95% Confidence Interval (CI) are estimated by Cox multivariable regression models.

|  | AS1 (N=66,722) | | AS2 (N=66,722) | | AS3 (N=63,456) | | AS4 (N=66,722) | |
| --- | --- | --- | --- | --- | --- | --- | --- | --- |
|  | HR [95% CI] | p-value | HR [95% CI] | p-value | HR [95% CI] | p-value | HR [95% CI] | p-value |
| Component 1, for 1 SD increase | 1.02 [0.97 - 1.08] | 0.421 | 1.01 [0.96 - 1.06] | 0.613 | 1.03 [0.97 - 1.09] | 0.309 | 1.02 [0.97 - 1.07] | 0.401 |
| Component 2, for 1 SD increase | 1.02 [0.97 - 1.06] | 0.495 | 1.02 [0.98 - 1.07] | 0.355 | 1.02 [0.98 - 1.07] | 0.324 | 1.03 [0.99 - 1.08] | 0.159 |
| Component 3, for 1 SD increase | 1.01 [0.97 - 1.04] | 0.763 | 1.01 [0.98 - 1.04] | 0.589 | 1.01 [0.97 - 1.04] | 0.753 | 1.01 [0.97 - 1.04] | 0.731 |
| Component 4, for 1 SD increase | 0.99 [0.96 - 1.03] | 0.642 | 0.99 [0.96 - 1.02] | 0.538 | 0.98 [0.95 - 1.02] | 0.349 | 0.99 [0.96 - 1.02] | 0.472 |
| Component 5, for 1 SD increase | 0.99 [0.95 - 1.04] | 0.784 | 1.00 [0.96 - 1.03] | 0.850 | 1.00 [0.97 - 1.04] | 0.868 | 1.00 [0.97 - 1.03] | 0.932 |

SD: standard deviation.

Use of age as the time-scale (years), stratification of the baseline hazard on birth generation (≤1930; (1930-1935]; (1935-1940]; (1940;1945]; >1945), and adjustment on school education level (<12 years; 12 to 14 years; >14 years), smoking status (non-smoker; former smoker; current smoker), body mass index (<18.5; [18.5-22.5); [22.5-25); [25-30); ≥30 kg/m2; missing values), parity and age at FFTP (nulliparous; one or two children and age at FFTP<30; more than two children and age at FFTP<30; age at FFTP≥30), cumulated duration of previous breastfeeding (no breastfeeding: less than 6 months of breastfeeding; at least 6 months of breastfeeding), utilization of contraceptive pill (ever; never), menopausal status and recent use of MHT (pre-menopaused, menopaused with recent use oh MHT; menopaused without recent use of MHT, menopaused and missing data on recent used of MHT), physical activity (continuously in metabolic equivalents of task-hour/week), daily alcohol intake (continuously in g of ethanol/day), daily lipids intake (continuously in g/day), daily total energy intake except from alcohol and lipid (continuously in kcal/day), and mutual adjustment of each principal component to each other.

AS1: Supplementary adjusted on adherence to western dietary pattern (continuously) and adherence to prudent dietary patterns (continuously).

AS2: Supplementary adjusted on adherence to French dietary guidelines (continuously).

AS3: Introduction of a 5-year lag between exposure assessment and the start of follow-up.

AS4: Use of a reduced set of adjustment, excluding the grey covariates.

# Supplementary table 6: Sensitivity analyses for the associations between principal components of POPs obtained by PLS-Cox and ER-positive breast cancer occurrence in the E3N cohort: additional adjustments on adherence to western and prudent dietary patterns (AS1), on adherence to French dietary guidelines (AS2), and introduction of a 5-year lag between exposure assessment and the start of follow-up (AS3). Hazard ratios (HR) and 95% Confidence Interval (CI) are estimated by Cox multivariable regression models.

|  | AS1 (N=20,127) | | AS2 (N=20,127) | | AS3 (N=19,156) | | AS4 (N=20,127) | |
| --- | --- | --- | --- | --- | --- | --- | --- | --- |
|  | HR [95% CI] | p-value | HR [95% CI] | p-value | HR [95% CI] | p-value | HR [95% CI] | p-value |
| Component 1, for 1 SD increase | 1.06 [0.96 - 1.17] | 0.258 | 1.06 [0.97 - 1.17] | 0.201 | 1.10 [0.99 - 1.23] | 0.069 | 1.07 [0.98 - 1.10] | 0.164 |
| Component 2, for 1 SD increase | 0.99 [0.92 - 1.06] | 0.751 | 0.99 [0.93 - 1.06] | 0.775 | 1.00 [0.94 - 1.08] | 0.927 | 1.00 [0.93 - 1.05] | 0.918 |
| Component 3, for 1 SD increase | 0.97 [0.91 - 1.03] | 0.311 | 0.97 [0.91 - 1.03] | 0.257 | 0.97 [0.91 - 1.04] | 0.389 | 0.97 [0.92 - 1.03] | 0.242 |
| Component 4, for 1 SD increase | 1.01 [0.96 - 1.07] | 0.668 | 1.01 [0.96 - 1.08] | 0.648 | 1.02 [0.96 - 1.09] | 0.550 | 1.01 [0.96 - 1.07] | 0.628 |
| Component 5, for 1 SD increase | 1.04 [0.96 - 1.11] | 0.331 | 1.05 [0.99 - 1.11] | 0.132 | 1.04 [0.97 - 1.11] | 0.272 | 1.05 [1.00 - 1.12] | 0.100 |

SD: standard deviation.

Use of age as the time-scale (years), stratification of the baseline hazard on birth generation (≤1930; (1930-1935]; (1935-1940]; (1940;1945]; >1945), and adjustment on school education level (<12 years; 12 to 14 years; >14 years), smoking status (non-smoker; former smoker; current smoker), body mass index (<18.5; [18.5-22.5); [22.5-25); [25-30); ≥30 kg/m2; missing values), parity and age at FFTP (nulliparous; one or two children and age at FFTP<30; more than two children and age at FFTP<30; age at FFTP≥30), cumulated duration of previous breastfeeding (no breastfeeding: less than 6 months of breastfeeding; at least 6 months of breastfeeding), utilization of contraceptive pill (ever; never), menopausal status and recent use of MHT (pre-menopaused, menopaused with recent use oh MHT; menopaused without recent use of MHT, menopaused and missing data on recent used of MHT), physical activity (continuously in metabolic equivalents of task-hour/week), daily alcohol intake (continuously in g of ethanol/day), daily lipids intake (continuously in g/day), daily total energy intake except from alcohol and lipid (continuously in kcal/day), and mutual adjustment of each principal component to each other.

AS1: Supplementary adjusted on adherence to western dietary pattern (continuously) and adherence to prudent dietary patterns (continuously).

AS2: Supplementary adjusted on adherence to French dietary guidelines (continuously).

AS3: Introduction of a 5-year lag between exposure assessment and the start of follow-up.

AS4: Use of a reduced set of adjustment, excluding the grey covariates.

# Supplementary table 7: Sensitivity analyses for the associations between principal components of POPs obtained by PLS-Cox and ER-positive breast cancer occurrence in the E3N cohort: modeling all continuous adjustment variables with penalized splines (AS4), and fitting the model on the entire original study sample (AS5). Hazard ratios (HR) and 95% Confidence Interval (CI) are estimated by Cox multivariable regression models.

|  | AS4 (N=20,127) | | AS5 (N=66,722) | |
| --- | --- | --- | --- | --- |
|  | HR [95% CI] | p-value | HR [95% CI] | p-value |
| Component 1, for 1 SD increase | 1.06 [0.97 - 1.17] | 0.210 | 1.03 [0.98 - 1.09] | 0.264 |
| Component 2, for 1 SD increase | 0.99 [0.93 - 1.06] | 0.793 | 1.03 [1.00 - 1.07] | 0.084 |
| Component 3, for 1 SD increase | 0.96 [0.91 - 1.02] | 0.238 | 1.02 [0.99 - 1.05] | 0.265 |
| Component 4, for 1 SD increase | 1.01 [0.96 - 1.07] | 0.680 | **1.05 [1.01 - 1.08]** | **0.006** |
| Component 5, for 1 SD increase | 1.05 [0.98 - 1.11] | 0.149 | 1.03 [0.99 - 1.06] | 0.142 |

SD: standard deviation.

Use of age as the time-scale (years), stratification of the baseline hazard on birth generation (≤1930; (1930-1935]; (1935-1940]; (1940;1945]; >1945), and adjustment on school education level (<12 years; 12 to 14 years; >14 years), smoking status (non-smoker; former smoker; current smoker), body mass index (<18.5; [18.5-22.5); [22.5-25); [25-30); ≥30 kg/m2; missing values), parity and age at FFTP (nulliparous; one or two children and age at FFTP<30; more than two children and age at FFTP<30; age at FFTP≥30), cumulated duration of previous breastfeeding (no breastfeeding: less than 6 months of breastfeeding; at least 6 months of breastfeeding), utilization of contraceptive pill (ever; never), menopausal status and recent use of MHT (pre-menopaused, menopaused with recent use oh MHT; menopaused without recent use of MHT, menopaused and missing data on recent used of MHT), physical activity (continuously, in metabolic equivalents of task-hour/week), daily alcohol intake (continuously, in g of ethanol/day), daily lipids intake (continuously, in g/day), and daily total energy intake except from alcohol and lipid (continuously, in kcal/day).

AS4: Modeling all continuous variables with penalized spline functions with 4 degrees of freedom.

AS5: Fitting the model on the entire study sample.

# Supplementary table 8: Reporting checklist for observational studies in nutritional epidemiology based on the STROBE-nut guidelines.

|  |  | Reporting Item | Page Number |
| --- | --- | --- | --- |
| **Title and abstract** | | |  |
| Title | [#1a](https://www.goodreports.org/reporting-checklists/strobe-nut/info/#1a) | Indicate the study’s design with a commonly used term in the title or the abstract | 1 |
| None | [#nut-1](https://www.goodreports.org/reporting-checklists/strobe-nut/info/#nut-1) | State the dietary/nutritional assessment method(s) used in the title or in the abstract. | 2 |
| Abstract | [#1b](https://www.goodreports.org/reporting-checklists/strobe-nut/info/#1b) | Provide in the abstract an informative and balanced summary of what was done and what was found | 2 |
| **Introduction** |  |  |  |
| Background / rationale | [#2](https://www.goodreports.org/reporting-checklists/strobe-nut/info/#2) | Explain the scientific background and rationale for the investigation being reported | 3-4 |
| Objectives | [#3](https://www.goodreports.org/reporting-checklists/strobe-nut/info/#3) | State specific objectives, including any prespecified hypotheses | 5 |
| **Methods** |  |  |  |
| Study design | [#4](https://www.goodreports.org/reporting-checklists/strobe-nut/info/#4) | Present key elements of study design early in the paper | 5 |
| Setting | [#5](https://www.goodreports.org/reporting-checklists/strobe-nut/info/#5) | Describe the setting, locations, and relevant dates, including periods of recruitment, exposure, follow-up, and data collection | 5,10-11 |
| None | [#nut-5](https://www.goodreports.org/reporting-checklists/strobe-nut/info/#nut-5) | Describe any characteristics of the study settings that might affect the dietary intake or nutritional status of the participants, if applicable. | 6 |
| Eligibility | [#6a](https://www.goodreports.org/reporting-checklists/strobe-nut/info/#6a) | Cohort study: Give the eligibility criteria and the sources and methods of selection of participants. Describe methods of follow-up. Case-control study: Give the eligibility criteria and the sources and methods of case ascertainment and control selection. Give the rationale for the choice of cases and controls. Cross-sectional study: Give the eligibility criteria, and the sources and methods of selection of participants. | 5,9 |
| None | [#nut-6](https://www.goodreports.org/reporting-checklists/strobe-nut/info/#nut-6) | Report any particular dietary, physiologic, or nutritional characteristics that were considered when selecting the target population. | n/a |
| None | [#6b](https://www.goodreports.org/reporting-checklists/strobe-nut/info/#6b) | Cohort study: For matched studies, give matching criteria and number of exposed and unexposed. Case-control study: For matched studies, give matching criteria and the number of controls per case. | n/a |
| Variables | [#7](https://www.goodreports.org/reporting-checklists/strobe-nut/info/#7) | Clearly define all outcomes, exposures, predictors, potential confounders, and effect modifiers. Give diagnostic criteria, if applicable | 6-8 |
| None | [#nut-7.1](https://www.goodreports.org/reporting-checklists/strobe-nut/info/#nut-7.1) | Clearly define foods, food groups, nutrients, or other food components (e.g., preparation method, taxonomical descriptors, classification, chemical form). | 5-6 |
| None | [#nut-7.2](https://www.goodreports.org/reporting-checklists/strobe-nut/info/#nut-7.2) | When calculating dietary patterns, describe the methods to obtain them and their nutritional properties. | 8 |
| Data sources and measurement | [#8](https://www.goodreports.org/reporting-checklists/strobe-nut/info/#8) | For each variable of interest give sources of data and details of methods of assessment (measurement). Describe comparability of assessment methods if there is more than one group. Give information separately for exposed and unexposed groups if applicable. | 6-8 |
| None | [#nut-8.1](https://www.goodreports.org/reporting-checklists/strobe-nut/info/#nut-8.1) | Describe the dietary assessment method(s) (e.g., portion size estimation, number of days and items recorded, how it was developed and administered, and how quality was ensured); report if and how supplement intake was assessed. | 5 |
| None | [#nut-8.2](https://www.goodreports.org/reporting-checklists/strobe-nut/info/#nut-8.2) | Describe and justify food-composition data used; explain the procedure to match food composition with consumption data; describe the use of conversion factors, if applicable | 5 |
| None | [#nut-8.3](https://www.goodreports.org/reporting-checklists/strobe-nut/info/#nut-8.3) | Describe the nutrient requirements, recommendations, or dietary guidelines and the evaluation approach used to compare intake with the dietary reference values, if applicable | n/a |
| None | [#nut-8.4](https://www.goodreports.org/reporting-checklists/strobe-nut/info/#nut-8.4) | When using nutritional biomarkers, additionally use the STROBE-ME; report the type of biomarkers used and usefulness as dietary exposure markers | n/a |
| None | [#nut-8.5](https://www.goodreports.org/reporting-checklists/strobe-nut/info/#nut-8.5) | Describe the assessment of nondietary data (e.g., nutritional status and influencing factors) and timing of the assessment of these variables in relation to dietary assessment | 8 |
| None | [#nut-8.6](https://www.goodreports.org/reporting-checklists/strobe-nut/info/#nut-8.6) | Report on the validity of the dietary or nutritional assessment methods and any internal or external validation used in the study, if applicable | 5 |
| Bias | [#9](https://www.goodreports.org/reporting-checklists/strobe-nut/info/#9) | Describe any efforts to address potential sources of bias | 12 |
| None | [#nut-9](https://www.goodreports.org/reporting-checklists/strobe-nut/info/#nut-9) | Report how bias in dietary or nutritional assessment was addressed (e.g., misreporting, changes in habits as a result of being measured, data imputation from other sources). | 9 |
| Study size | [#10](https://www.goodreports.org/reporting-checklists/strobe-nut/info/#10) | Explain how the study size was arrived at | 9 |
| Quantitative variables | [#11](https://www.goodreports.org/reporting-checklists/strobe-nut/info/#11) | Explain how quantitative variables were handled in the analyses. If applicable, describe which groupings were chosen, and why | 11 |
| None | [#nut-11](https://www.goodreports.org/reporting-checklists/strobe-nut/info/#nut-11) | Explain categorization of dietary/nutritional data (e.g., use of N-tiles and handling of nonconsumers) and the choice of reference category, if applicable. | n/a |
| Statistical methods | [#12a](https://www.goodreports.org/reporting-checklists/strobe-nut/info/#12a) | Describe all statistical methods, including those used to control for confounding | 9-11 |
| Subgroups and interactions | [#12b](https://www.goodreports.org/reporting-checklists/strobe-nut/info/#12b) | Describe any methods used to examine subgroups and interactions | n/a |
| Missing data | [#12c](https://www.goodreports.org/reporting-checklists/strobe-nut/info/#12c) | Explain how missing data were addressed | 8 |
| Loss to follow up | [#12d](https://www.goodreports.org/reporting-checklists/strobe-nut/info/#12d) | Cohort study: if applicable, explain how loss to follow-up was addressed. Case-control study: if applicable, explain how matching of cases and controls was addressed. Cross-sectional study: if applicable, describe analytical methods taking account of sampling strategy. | 10 |
| Sensitivity analysis | [#12e](https://www.goodreports.org/reporting-checklists/strobe-nut/info/#12e) | Describe any sensitivity analyses | 12 |
| None | [#nut-12.1](https://www.goodreports.org/reporting-checklists/strobe-nut/info/#nut-12.1) | Describe any statistical method used to combine dietary or nutritional data, if applicable. | n/a |
| None | [#nut-12.2](https://www.goodreports.org/reporting-checklists/strobe-nut/info/#nut-12.2) | Describe and justify the method for energy adjustments, intake modeling, and use of weighting factors, if applicable | 11 |
| None | [#nut-12.3](https://www.goodreports.org/reporting-checklists/strobe-nut/info/#nut-12.3) | Report any adjustments for measurement error (i.e., from a validity or calibration study). | n/a |
| **Results** |  |  |  |
| Participants | [#13a](https://www.goodreports.org/reporting-checklists/strobe-nut/info/#13a) | Report numbers of individuals at each stage of study—eg numbers potentially eligible, examined for eligibility, confirmed eligible, included in the study, completing follow-up, and analysed. Give information separately for for exposed and unexposed groups if applicable. | 12 |
| Non-participation | [#13b](https://www.goodreports.org/reporting-checklists/strobe-nut/info/#13b) | Give reasons for non-participation at each stage | 12 |
| Participant journey | [#13c](https://www.goodreports.org/reporting-checklists/strobe-nut/info/#13c) | Consider the use of a flow diagram | n/a |
| None | [#nut-13](https://www.goodreports.org/reporting-checklists/strobe-nut/info/#nut-13) | Report the number of individuals excluded on the basis of missing, incomplete, or implausible dietary and nutritional data. | 12 |
| Descriptive data | [#14a](https://www.goodreports.org/reporting-checklists/strobe-nut/info/#14a) | Give characteristics of study participants (eg demographic, clinical, social) and information on exposures and potential confounders. Give information separately for exposed and unexposed groups if applicable. | 12, Table 1 |
| Missing data | [#14b](https://www.goodreports.org/reporting-checklists/strobe-nut/info/#14b) | Indicate number of participants with missing data for each variable of interest | 12, Table 1 |
| Follow-up time | [#14c](https://www.goodreports.org/reporting-checklists/strobe-nut/info/#14c) | Cohort study: Summarise follow-up time (eg, average and total amount) | 12 |
| None | [#nut-14](https://www.goodreports.org/reporting-checklists/strobe-nut/info/#nut-14) | Give the distribution of participant characteristics across the exposure variables, if applicable; specify if food consumption for the total population or consumers only was used to obtain results | n/a |
| Outcome data | [#15](https://www.goodreports.org/reporting-checklists/strobe-nut/info/#15) | Cohort study: report numbers of outcome events or summary measures over time. Case-control study: report numbers in each exposure category, or summary measures of exposure. Cross-sectional study: report numbers of outcome events or summary measures. | 12 |
| Main results | [#16a](https://www.goodreports.org/reporting-checklists/strobe-nut/info/#16a) | Give unadjusted estimates and, if applicable, confounder-adjusted estimates and their precision (eg, 95% confidence interval). Make clear which confounders were adjusted for and why they were included | 13-14, Tables 2, 3 ,4 |
| Category boundaries | [#16b](https://www.goodreports.org/reporting-checklists/strobe-nut/info/#16b) | Report category boundaries when continuous variables were categorized | Tables 2, 3, 4 |
| Relative and absolute risks | [#16c](https://www.goodreports.org/reporting-checklists/strobe-nut/info/#16c) | If relevant, consider translating estimates of relative risk into absolute risk for a meaningful time period | n/a |
|  |  |  |  |
| None | [#nut-16](https://www.goodreports.org/reporting-checklists/strobe-nut/info/#nut-16) | Specify if nutrient intakes are reported with or without the inclusion of dietary supplement intake, if applicable. | n/a |
| Other analyses | [#17](https://www.goodreports.org/reporting-checklists/strobe-nut/info/#17) | Report other analyses done—eg analyses of subgroups and interactions, and sensitivity analyses | 14 |
| None | [#nut-17](https://www.goodreports.org/reporting-checklists/strobe-nut/info/#nut-17) | Report any sensitivity analysis (e.g., exclusion of misreporters or outliers) and data imputation, if applicable | 14 |
| **Discussion** |  |  |  |
| Key results | [#18](https://www.goodreports.org/reporting-checklists/strobe-nut/info/#18) | Summarise key results with reference to study objectives | 14-15 |
| Limitations | [#19](https://www.goodreports.org/reporting-checklists/strobe-nut/info/#19) | Discuss limitations of the study, taking into account sources of potential bias or imprecision. Discuss both direction and magnitude of any potential bias. | 16-17 |
| None | [#nut-19](https://www.goodreports.org/reporting-checklists/strobe-nut/info/#nut-19) | Describe the main limitations of the data sources and assessment methods used and implications for the interpretation of the findings | 16-17 |
| Interpretation | [#20](https://www.goodreports.org/reporting-checklists/strobe-nut/info/#20) | Give a cautious overall interpretation considering objectives, limitations, multiplicity of analyses, results from similar studies, and other relevant evidence. | 15-16 |
| None | [#nut-20](https://www.goodreports.org/reporting-checklists/strobe-nut/info/#nut-20) | Report the nutritional relevance of the findings, given the complexity of diet or nutrition as an exposure. | 15-16 |
| Generalisability | [#21](https://www.goodreports.org/reporting-checklists/strobe-nut/info/#21) | Discuss the generalisability (external validity) of the study results | 15-16 |
| **Other Information** | |  |  |
| Funding | [#22](https://www.goodreports.org/reporting-checklists/strobe-nut/info/#22) | Give the source of funding and the role of the funders for the present study and, if applicable, for the original study on which the present article is based | 25 |
| Ethics | [#nut-22.1](https://www.goodreports.org/reporting-checklists/strobe-nut/info/#nut-22.1) | Describe the procedure for consent and study approval from ethics committee(s). | 25 |
| Data statement | [#nut-22.2](https://www.goodreports.org/reporting-checklists/strobe-nut/info/#nut-22.2) | Provide data collection tools and data as online material or explain how they can be accessed | 25 |

n/a: not applicable

The STROBE-nut checklist is distributed under the terms of the Creative Commons Attribution License CC-BY. This checklist can be completed online using <https://www.goodreports.org/>, a tool made by the [EQUATOR Network](https://www.equator-network.org) in collaboration with <https://www.penelope.ai>

# Annex 1: Formulas of the main Cox models fitted for each of the three approaches (Varclus-Cox, PCR-Cox and PLS-Cox) used to estimate the associations between dietary exposures to combinations of POPs and ER-positive breast cancer risk in the French E3N cohort study.

The formulas are presented for the final Cox models fitted after identifying the final numbers of clusters and principal components.

Varclus-Cox

λ(Age|**Covar**)= λ_0Gene_(Age) * exp(β_1_Clus1 + β_2_Clus2 + β_3_Clus3 + β_4_Clus4 + β_5_Clus5 + β_6_Clus6 + β_7_Clus7 + β_8_Clus8 + β_9_Clus9 + β_10_Clus10 + β_11_Clus11 + β_12_Etu1 + β_13_Etu2 + β_14_Smoke1 + β_15_Smoke 2 + β_16_BMI1 + β_17_BMI2 + β_18_BMI3 + β_19_BMI4 + β_20_BMI5 + β_22_Physact + β_23_ParityAge1 + β_23_ParityAge2 + β_24_ParityAge3 + β_25_Breastfeed1 + β_26_Breastfeed2 + β_27_ContraceptivePill + β_28_MenoMHT1 + β_29_MenoMHT2 + β_30_MenoMHT3 + β_31_Alcohol + β_32_Lipids + β_33_Energy)

PCR-Cox

λ(Age|**Covar**)= λ_0Gene_(Age) * exp(β_1_CompPCA1 + β_2_ CompPCA2 + β_3_ CompPCA3 + β_4_ CompPCA4 + β_5_CompPCA5 + β_6_Etu1 + β_7_Etu2 + β_8_Smoke1 + β_9_Smoke 2 + β_10_BMI1 + β_11_BMI2 + β_12_BMI3 + β_13_BMI4 + β_14_BMI5 + β_15_Physact + β_16_ParityAge1 + β_17_ParityAge2 + β_18_ParityAge3 + β_19_Breastfeed1 + β_20_Breastfeed2 + β_21_ContraceptivePill + β_22_MenoMHT1 + β_23_MenoMHT2 + β_24_MenoMHT3 + β_25_Alcohol + β_26_Lipids + β_27_Energy)

PLS-Cox

λ(Age|**Covar**)= λ_0Gene_(Age) * exp(β_1_CompPLS1 + β_2_ CompPLS2 + β_3_ CompPLS3 + β_4_ CompPLS4 + β_5_CompPCA5 + β_6_Etu1 + β_7_Etu2 + β_8_Smoke1 + β_9_Smoke 2 + β_10_BMI1 + β_11_BMI2 + β_12_BMI3 + β_13_BMI4 + β_14_BMI5 + β_15_Physact + β_16_ParityAge1 + β_17_ParityAge2 + β_18_ParityAge3 + β_19_Breastfeed1 + β_20_Breastfeed2 + β_21_ContraceptivePill + β_22_MenoMHT1 + β_23_MenoMHT2 + β_24_MenoMHT3 + β_25_Alcohol + β_26_Lipids + β_27_Energy)

**Legends**

λ(Age|**Covar**): Hazard function modelling the instantaneous risk of developing estrogen-receptor positive breast cancer at a given age, depending on the covariates vector.

λ_0Gene_(Age): Baseline hazard function depending on the birth generation, taking a different value for each category: ≤1930; (1930-1935]; (1935-1940]; (1940;1945]; >1945.

Clus1 to Clus11: Summary statistics for clusters 1 to 11 identified by hierarchical clustering.

CompPCA1 to CompPCA5: Principal components 1 to 5 identified by principal component analysis.

CompPLS1 to CompPCA5: Principal components 1 to 5 identified by partial least square Cox regression.

Etu1 to Etu2: Dummies variables for school education level.

|  | School education level | | |
| --- | --- | --- | --- |
|  | <12 years | 12 to 14 years | >14 years |
| Etu1 | 0 | 1 | 0 |
| Etu2 | 0 | 0 | 1 |

Smoke1 to Smoke2: Dummies variables for smoking status.

|  | Smoking status | | |
| --- | --- | --- | --- |
|  | Non-smoker | Former smoker | Current smoker |
| Smoke1 | 0 | 1 | 0 |
| Smoke2 | 0 | 0 | 1 |

BMI1 to BMI5: Dummies variables for body mass index.

|  | Body mass index in kg/m^2^ | | | | | |
| --- | --- | --- | --- | --- | --- | --- |
|  | <18.5 | [18.5-22.5) | [22.5-25) | [25-30) | ≥30 | Missing value |
| BMI1 | 0 | 1 | 0 | 0 | 0 | 0 |
| BMI2 | 0 | 0 | 1 | 0 | 0 | 0 |
| BMI3 | 0 | 0 | 0 | 1 | 0 | 0 |
| BMI4 | 0 | 0 | 0 | 0 | 1 | 0 |
| BMI5 | 0 | 0 | 0 | 0 | 0 | 1 |

Physact: Physical activity (continuously in metabolic equivalents of task-hour/week).

ParityAge1 to ParityAge3: Dummies variables for parity and age at first full-term pregnancy (FFTP).

|  | Parity and age at first full-term pregnancy | | | |
| --- | --- | --- | --- | --- |
|  | Nulliparous | 1-2 children and age at FFTP<30 | >2 children and age at FFTP<30 | age at FFTP≥30 |
| ParityAge1 | 0 | 1 | 0 | 0 |
| ParityAge2 | 0 | 0 | 1 | 0 |
| ParityAge3 | 0 | 0 | 0 | 1 |

Breastfeed1 to Breastfeed2: Dummies variables for cumulated duration of previous breastfeeding.

|  | Cumulated duration of previous breastfeeding | | |
| --- | --- | --- | --- |
|  | No breastfeeding | Less than 6 months | At least 6 months |
| Breastfeed1 | 0 | 1 | 0 |
| Breastfeed2 | 0 | 0 | 1 |

ContraceptivePill: Ever use of contraceptive pill.

MenoMHT1 to MenoMHT3: Menopausal status and recent use of menopausal hormone therapy (MHT) (pre-menopaused, menopaused with recent use oh MHT; menopaused without recent use of MHT, menopaused and missing data on recent used of MHT).

|  | Menopausal status and recent use of MHT | | | |
| --- | --- | --- | --- | --- |
|  | Pre-menopaused | Menopaused with recent use of MTH | Menopaused without recent use oh MHT | Menopaused and missing data of recent use of MHT |
| MenoMHT1 | 0 | 1 | 0 | 0 |
| MenoMHT2 | 0 | 0 | 1 | 0 |
| MetoMHT3 | 0 | 0 | 0 | 1 |

Alcohol: daily lipids intake (continuously in g/day).

Lipids: daily alcohol intake (continuously in g of ethanol/day).

Energy: daily total energy intake except from alcohol and lipid (continuously in kcal/day).
